# TNFR2 Is a Crucial Hub Controlling Mesenchymal Stem Cell Biological and Functional Properties

**DOI:** 10.3389/fcell.2020.596831

**Published:** 2020-12-04

**Authors:** Ghada Beldi, Sheyda Bahiraii, Chloé Lezin, Mahsa Nouri Barkestani, Mohamed Essameldin Abdelgawad, Georges Uzan, Sina Naserian

**Affiliations:** ^1^INSERM UMR-S-MD 1197, Hôpital Paul Brousse, Villejuif, France; ^2^Department of Pharmacognosy, University of Vienna, Vienna, Austria; ^3^Paris-Saclay University, Villejuif, France; ^4^Biochemistry Division, Chemistry Department, Faculty of Science, Helwan University, Cairo, Egypt; ^5^CellMedEx, Saint-Maur-Des-Fossés, France

**Keywords:** mesenchymal stem cells, immunoregulation, immune-checkpoint, tissue regeneration, angiogenesis, TNFα–TNFR2 signaling pathway

## Abstract

Mesenchymal stem cells (MSCs) have drawn lots of attention as gold standard stem cells in fundamental and clinical researches during the last 20 years. Due to their tissue and vascular repair capacities, MSCs have been used to treat a variety of degenerative disorders. Moreover, MSCs are able to modulate immune cells’ functions, particularly T cells while inducing regulatory T cells (iTregs). MSCs are very sensitive to inflammatory signals. Their biological functions could remarkably vary after exposure to different pro-inflammatory cytokines, notably TNFα. In this article, we have explored the importance of TNFR2 expression in a series of MSCs’ biological and functional properties. Thus, MSCs from wild-type (WT) and TNFR2 knockout (TNFR2 KO) mice were isolated and underwent several *ex vivo* experiments to investigate the biological significance of TNFR2 molecule in MSC main functions. Hampering in TNFR2 signaling resulted in reduced MSC colony-forming units and proliferation rate and diminished the expression of all MSC characteristic markers such as stem cell antigen-1 (Sca1), CD90, CD105, CD44, and CD73. TNFR2 KO-MSCs produced more pro-inflammatory cytokines like TNFα, IFNγ, and IL-6 and less anti-inflammatory mediators such as IL-10, TGFβ, and NO and induced Tregs with less suppressive effect. Furthermore, the TNFR2 blockade remarkably decreased MSC regenerative functions such as wound healing, complex tube formation, and endothelial pro-angiogenic support. Therefore, our results reveal the TNFα–TNFR2 axis as a crucial regulator of MSC immunological and regenerative functions.

## Introduction

In 1968, for the first time, a heterogeneous population of bone marrow (BM)-derived stem cells were identified and introduced to the scientific world, referred to as mesenchymal stem/stromal cells (MSCs) (Friedenstein et al., 1968). These cells bear different important potentials like self-renewal and proliferation and can differentiate into multilineage cell types of different origins such as endodermal, ectodermal, and mesodermal including (but not limited to) myocytes, cardiomyocytes, neural cells, hepatocytes and endothelial cells (ECs), adipocytes, osteocytes, and chondrocytes (Han et al., 2017; Khosravi et al., 2018a; Afshari et al., 2020; Zhang et al., 2020).

MSCs demonstrated three principal biological characteristics that make them specifically interesting for cell therapy applications: (1) differentiation potential, (2) secretion of different mediators, and (3) immunoregulatory properties (Khosravi et al., 2017, 2018b,c; Beldi et al., 2020). Based on these features, in addition to their ability to be simply expanded and manipulated *ex vivo*, MSC fundamental and clinical research has evolved rapidly during the last decade.

Despite our advanced knowledge, there currently exist no distinctive markers to phenotypically identify and characterize MSCs or their subpopulations. However, depending on their origin species, they are expected to express stem cell antigen-1 (Sca1), CD44, CD105, CD73, and CD90 and must not express CD14 monocyte, CD34 hematopoietic, and CD45 common leukocyte markers (Dominici et al., 2006; Maleki et al., 2014).

MSCs are sensitive and responsive to microenvironment changes. Their biological properties are strongly influenced by the interaction with other cells, extracellular matrix, soluble bioactive factors like cytokines, and most importantly the presence and intensity of the surrounding inflammation (Chen et al., 2015; Broekman et al., 2016; Daneshmandi et al., 2017; Zhou et al., 2017; Bai et al., 2018; Noronha et al., 2019). The modulation of several biological and/or biochemical elements has been proven to augment MSC therapeutic capacity through impacting MSC’s fate, differentiation, and function (Nava et al., 2012; Huang et al., 2015). Phenotypical profile and immunomodulatory functions of MSCs could also be altered by exposure to an inflamed environment (Pourgholaminejad et al., 2016). Many studies previously evidenced that treating MSCs with pro-inflammatory cytokines like IL-1β, IL-17, IFNγ, and TNFα could modify their differentiation, proliferation, migration, immunomodulation, and cytokine production capacities via a range of signaling pathways particularly NF-κB and STAT3 activation (Noronha et al., 2019). Priming enhances MSC anti-inflammatory and immunosuppressive functions via upregulating important immunoregulatory elements, such as IDO, PGE2, hepatocyte growth factor (HGF), CCL2, TGFβ, and IL-10 (Prasanna et al., 2010; de Witte et al., 2015; Baldari et al., 2017; Vigo et al., 2017; Bai et al., 2018; Hu and Li, 2018; Noronha et al., 2019). Moreover, TNFα pre-treatment increases MSC ability to induce monocyte differentiation into IL-10 secreting immunosuppressive M2 macrophages (François et al., 2012). Equally, primed MSCs have enhanced T cell immunosuppressive effect that is associated with increased nitric oxide (NO) production, a major murine MSC immunosuppressive molecule (Szabó et al., 2015; Pourgholaminejad et al., 2016; Noronha et al., 2019).

By binding to its receptors (TNFR1 and TNFR2), TNFα exerts miscellaneous and complex biological activities. Per contra to TNFR1 ubiquitous expression on every cell, TNFR2 has rather restricted expression notably by ECs, neural cells, immune cells, and MSCs (Kelly et al., 2010; Chen and Palmer, 2013; Salomon et al., 2018; Yang S. et al., 2018). Even though TNFα recognizes both receptors, its interaction with either of them could end in an entirely different biological outcome.

Previous researches on MSCs established that the TNFα–TNFR2 signaling pathway leads to pro-angiogenic, protective, and survival mechanisms, while TNFα–TNFR1 signaling generally terminates in harmful and pro-apoptotic mechanisms (Faustman and Davis, 2013). For instance, administration of BM MSCs (BM-MSCs) harvested from TNFR2 knockout (KO) mice in acute ischemia model displayed impaired or no myocardial recovery function, which was followed by elevated pro-inflammatory factors and reduced vascular endothelial growth factor (VEGF) production (Kelly et al., 2010; Tan et al., 2010). Accordingly, other studies showed that TNFα primed human BM-MSCs have enhanced capacity to produce important growth factors such as HGF, insulin-like growth factor 1 (IGF-1), and VEGF via a TNFR2-dependent mechanism (Crisostomo et al., 2008; Zhang et al., 2010). Besides, TNFR2 upregulation in rat and human BM-MSCs effectively increased their therapeutic potential in cardiac ischemia (Bao et al., 2008, 2010) and inflammatory disorders like rheumatoid arthritis (RA), which was accompanied by decreased IL-6, IL-1β, and TNFα secretion (Liu et al., 2013; Park et al., 2017).

Our cumulative research works from the last decade demonstrate a straight association between the TNFR2 expression and the immunomodulatory properties of different cells including regulatory T cells (Tregs), myeloid-derived suppressor cells (MDSCs), Bregs, MSCs, and endothelial progenitor cells (EPCs) (Polz et al., 2014; Leclerc et al., 2016; Ticha et al., 2017; Beldi et al., 2020; Naserian et al., 2020). Accordingly, the same phenomenon was speculated for neural stem/progenitor cells, which are the other subcategory of rare TNFR2-expressing cells (Shamdani et al., 2020).

Recently, in an attempt to investigate the immunological importance of TNFR2 expression on MSCs and to evaluate its role in MSC/T cell cross talk, we revealed TNFα–TNFR2 as an indispensable immune checkpoint signaling pathway involved in MSC immunomodulatory effect against T cells. We have reported that unlike TNFR2 KO BM-MSCs, their TNFR2-expressing wild-type (WT) counterparts were remarkably more efficient in T cell suppression, down-modulating T cell activation markers, reducing T cell pro-inflammatory factors, and increasing their anti-inflammatory cytokine production. Moreover, blocking TNFR2 signaling caused a significant decrease in MSC ability to convert conventional T cells (Tconvs) to induced Tregs (iTregs) (Beldi et al., 2020).

Here, in this complementary study, we emphasize more specifically on the impact of TNFR2 expression on a series of MSC biological and functional properties that have not been revealed yet. Therefore, we aimed to investigate (1) if MSCs from WT or TNFR2 KO mice have different morphological, colony forming, proliferation, and differentiation properties; (2) if MSCs from WT or TNFR2 KO mice express different levels of characteristic markers; (3) if the absence of TNFR2 could alter MSC cytokine secretion and NO production pattern and eventually; and (4) if Tregs induced from WT and TNFR2 KO mice have different immunosuppressive efficiency. Concerning the indispensable MSC regenerative properties, we have evaluated the impact of TNFR2 blockade on (1) MSC wound healing capability of a scratched monolayer, (2) MSC ability to form complex 3D tubular structures on extracellular matrix component, and finally (3) MSC ability to support the pro-angiogenic function of human umbilical vein ECs (HUVECs).

## Materials and Methods

### Mesenchymal Stem Cell Isolation

MSCs were isolated from total BM harvested from femurs and tibias of 6–8 weeks old C57BL/6 WT (Charles River and Envigo) and C57BL/6 TNFR2 KO mice (B6.129S2-Tnfrsf1b^tm1Mwm^ /J, The Jackson Laboratory) housed under pathogen-free conditions as already described (Beldi et al., 2020). The TNFR2 KO mice did not demonstrate any different phenotypes as compared with WT mice. They had normal lymphoid tissues and did not develop any spontaneous pathology. Harvested cells were cultured in 25 cm^2^ flasks in Dulbecco’s modified Eagle’s medium (DMEM) medium (Gibco) supplemented with 1% GlutaMAX, 10% fetal bovine serum (FBS) (Eurobio, ref CVFSVF00-01), and 1% penicillin–streptomycin–neomycin (P/S/N) (Gibco), hereinafter referred to as complete DMEM and incubated at 37°C in 5% CO_2_. Every 8 h, non-adherent cells were eliminated, and the remaining cells were washed carefully with phosphate-buffered saline (PBS) 1×, and then the fresh complete medium was added. The initial colonies of MSCs (passage 0) appeared after 4–5 weeks of culture. The best and purest colonies according to their morphological appearance, homogeneity, and the speed of colony growth were selected and detached using cell dissociation reagent TrypLE (Gibco). From passage 1, cells were seeded at 5,000 cells/cm^2^. After the expansion phase, all cells were evaluated for the expression of MSC phenotypical characterization markers as explained in the next section. Furthermore, MSCs underwent a series of quality controls for evaluation of their functional features, such as colony-forming unit (CFU), immunosuppression, and differentiation capacities. Merely, the qualified cells were passed and/or stored at −150°C for further experimental procedures. Passages 1–3 of WT and TNFR2 KO-MSCs were used throughout the experiments depending on the experimental requirements.

### Mesenchymal Stem Cell Phenotypical Characterization

A total of 10^5^ WT and TNFR2 KO-MSCs were seeded in 96-well round-bottom plates (Falcon). Thereafter, they were immunostained using the following Abs: CD44-PE-Vio770, CD105-FITC, Sca1-APC, CD73-PE, CD90-Biotin or PE, anti-biotin-PE or VIOBLUE, CD45-VIOBLUE, CD34-Biotin or FITC (Miltenyi), and streptavidin-PeCY5/CY7 (eBioscience). We used isotypes Abs (Miltenyi) and unstained MSCs as controls for our experiments.

For F-actin staining, we have used F-actin Staining Kit, Green Fluorescence, Cytopainter (Abcam) according to supplier’s protocol. Briefly, we have seeded 10^5^ WT and TNFR2 KO MSCs in 96-well plates, treated them with 100 μl/well of Green Fluorescent Phalloidin Conjugate solution, and stained them in room temperature for 30 min. We then washed the cells with PBS 1× and analyzed in FITC channel using LSRFORTESSA flow cytometer (BD Biosciences). Data were then analyzed using FlowJo software v10 (FlowJo, LLC).

### Mesenchymal Stem Cell Colony Forming Unit Assays

A total of 200 WT or TNFR2 KO-MSCs (P1) were seeded in 25 cm^2^ flasks in 5 ml of complete DMEM. After 7 days, culture media were changed, and flasks were re-incubated for another 3 days. The staining solution was prepared with a crystal violet solution (Sigma Aldrich) diluted 1/20 with osmosis water. Culture media were removed, and flasks were rinsed twice with PBS 1× without calcium and magnesium. Staining solution was added (3 ml/flask) and left for 20 min at room temperature. Then the flasks were rinsed twice with osmosis water and left to dry. To count the number of CFUs, high-resolution pictures were taken and analyzed in a double-blinded manner using Image J software (National Institutes of Health, Bethesda, MD, United States).

### Mesenchymal Stem Cell Proliferation Measurement

WT and TNFR2 KO-MSCs from different passages (i.e., P1, P2, and P3) were seeded in 6-well plates (Falcon) with an initial density of 3 × 10^4^ cells/well. Cells were cultured in complete DMEM at 37°C and 5% CO_2_. The cell culture medium was changed once every other day for a duration of 5 days. Every 24 h cells were detached using TrypLE (Gibco). We determined the cell numbers with an automated cell counter apparatus (Countess II). Cell population doubling time (CPDT) was calculated using the following formula: CPDT = (t − t0) × log2 × (log[N/N0]) − 1, with t as time and N as cell number, according to already published articles (Zhou et al., 2008). Data were analyzed using FlowJo software v10 (FlowJo, LLC).

### Mesenchymal Stem Cell Viability Measurement

A total of 2 × 10^5^ WT or TNFR2 KO-MSCs (P1) were seeded in 25-cm^2^ flasks. Cells were cultured untreated or after a pre-treatment with 0.1, 1, 5, 10, and 100 ng/ml of premium-grade mouse TNFα (Miltenyi) for a duration of 24 h. Then, they were detached using cell dissociation reagent TrypLE (Gibco). The percentage of viable cells was determined by the absence of the expression of annexin V and propidium iodide (PI) markers using an annexin V-FITC kit (Miltenyi) with respect to the supplier’s protocol. Events were acquired on a LSRFORTESSA flow cytometer (BD Biosciences) and analyzed using FlowJo software v10 (FlowJo, LLC).

### Mesenchymal Stem Cell Differentiation Assay

The induction of adipogenic differentiation was performed by culturing the cells in a homemade adipogenic differentiation medium for 21 days as already described (Doucet et al., 2005). Thereafter, we stained the cells using Oil Red for a duration of 5 min. For osteocyte differentiation, we purchased a specific medium containing P/S at 1:100 dilution + StemXVIVO Mouse/Rat Osteogenic supplement 20 × (R&D Systems). WT and TNFR2 KO-MSCs were cultured for 17 days. Then, they were stained for 3 min using 2% Alizarin red. Samples were visualized by the objectives of 4 × and 10 × of the microscope (EVOS XL Core, Invitrogen by Thermo Fisher Scientific).

### Mesenchymal Stem Cell Cytokine Quantification

A total of 5 × 10^5^ WT or TNFR2 KO-MSCs were seeded in 75 cm^2^ flasks and were activated by the addition of 10 ng/ml of premium-grade mouse TNFα (Miltenyi) in a total volume of 10 ml. After 36 h, MSCs were treated with 1 μl/ml of Golgi-Plug (protein transport inhibitor) (BD Biosciences) and incubated for another 12 h. Cells were then stained with following Abs: CD44-PE-Vio770, CD73-PE, IFNγ-APC, IL-6-PE or FITC, TNFα-FITC, IL-10-APC (Miltenyi), and anti-TGFβ-PE (BioLegend). Events were acquired on a LSRFORTESSA flow cytometer (BD Biosciences) and analyzed using FlowJo software v10 (FlowJo, LLC).

### T Cell Isolation

T cell isolation was performed as previously reported (Beldi et al., 2020). Briefly, we utilized mouse Pan T Cell Isolation kit (Miltenyi) in order to separate CD3^+^ T cells from pooled lymph nodes and spleens of 6–12 weeks old WT C57BL/6 female mice (Charles River and Envigo). CD25^+^ subpopulation was depleted from the total CD3^+^ T cells by means of the anti-CD25 biotin-conjugated antibody (BD Biosciences), tailed by anti-biotin microbeads immunostaining (Miltenyi). Afterward, magnetic-activated cell sorting (MACS) was used for the cell isolation process. The sorted CD3^+^CD25^–^ T cells with higher than 94% purity were co-cultured with WT or TNFR2 KO-MSCs.

### Co-culture of Mesenchymal Stem Cells and T Cells

The co-culture of MSCs with T cells was performed as previously reported (Beldi et al., 2020). Briefly, WT or TNFR2 KO-MSCs were seeded either into 75 cm^2^ flasks or into 6 or 12-well plates and incubated for 6–24 h, in complete DMEM. Then, depending on the experimental conditions, different ratios of CD3^+^CD25^–^ T cells were added in Roswell Park Memorial Institute (RPMI) medium supplemented with 10% FBS (Eurobio, ref CVFSVF00-01), 1% P/S/N, 1% HEPES, and 5 × 10^–5^ M of β-mercaptoethanol, hereinafter referred to as complete RPMI. All co-culture experiments were performed in a mixture of 50% DMEM and 50% RPMI media. Suspending T cells were collected by gentle aspiration based on MSC’s ability to adhere to plastic.

### Regulatory T Cell Induction and Mixed Lymphocyte Reaction Assay

Freshly isolated CD3^+^CD25^–^ mouse T cells were activated by mouse anti-CD3/CD28 beads (Dynabeads, Gibco) with respect to the supplier’s protocol. A total of 5 × 10^5^ WT or TNFR2 KO-MSCs were seeded in 75 cm^2^ flasks for a duration of 6 h. Afterward, they were co-cultured with 5 × 10^6^ of bead-activated mouse CD3^+^CD25^–^ T cells (1/10 MSC/T cell ratio) in a final volume of 10 ml using a mixture of 50% RPMI and 50% DMEM media. We have used 5 × 10^5^ non-activated and activated CD3^+^CD25^–^ mouse T cells cultured alone as negative and positive controls, respectively. After 72 h, the CD4^+^CD25^+^ mouse regulatory T cell isolation kit was utilized to isolate iTregs according to the manufacturer’s instructions (Miltenyi). The purity of CD4^+^CD25^+^ iTregs (more than 90%) was determined via flow cytometric analysis using the following Abs: CD4-VIOBLUE, CD25-PE-Vio770, CTLA4-PE, TNFR2-APC (Miltenyi), and Foxp3-PE-Cy5 (eBioscience). A total of 2 × 10^4^ iTregs were then set in a mixed lymphocyte reaction (MLR) assay in a 96-well plate with 10^5^ freshly isolated activated CSFE^+^CD3^+^CD25^–^ responder T cells (Tconvs) in a fixed 1/5 iTreg/Tconv ratio. Cells were kept in a final volume of 200 μl of complete RPMI medium at 37°C in 5% CO_2_. We used 10^5^ non-activated and activated mouse CFSE^+^CD3^+^CD25^–^ T cells alone as negative and positive controls, respectively. After 72 h, we collected the cells and immunostained them using CD4-VIOBLUE and CD8α-PE-Vio770 antibodies. The percentage of proliferating CD4^+^ and CD8^+^ T cells was analyzed by measuring the carboxyfluorescein succinimidyl ester (CFSE) dilution using LSRFORTESSA flow cytometer (BD Biosciences). Data were then analyzed using FlowJo software v10 (FlowJo, LLC).

### Nitric Oxide Measurement

Nitric oxide was quantified using DAF-2/DA probe according to supplier’s instructions (Eugene, Oregon, United States). A total of 10^5^ MSCs were seeded into 12-well plates and incubated with 5 μM of DAF-2/DA in complete DMEM medium containing 10% FBS or in DMEM medium containing 0.5% FBS for a duration of 1 h at 37°C. Plates were kept away from light exposure during the experiment. Cells were washed to remove excess probe and detached using cell dissociation reagent TrypLE (Gibco). Thereafter, cells were re-suspended in PBS 1× supplemented with 0.5% bovine serum albumin (BSA) and immediately detected in FITC channel using LSRFORTESSA flow cytometer (BD Biosciences) and analyzed by FlowJo software v10 (FlowJo, LLC).

### Wound Healing Assay

Wound healing assays were conducted by means of the specific culture insert with respect to the supplier’s instructions (IBIDI, Germany). WT and TNFR2 KO-MSCs were seeded in the two separate wells of the insert at 15 × 10^3^ cells/ml in a final volume of 70 μl/well, to acquire a confluent layer within 24 h. To create a 500 μm homogeneous cell-free gap, culture inserts were carefully removed. The wounded area appearance was instantaneously captured by taking pictures (Nikon D3100, Japan) using objectives 4× and 10× of the microscope (Nikon ECLIPSE T5100) and continued every 2 h, until complete closure in WT-MSC group. Cells were incubated at 37°C in 5% CO_2_. The decrease in wounded area was analyzed by measuring the scratch area size using the plugin MRI Wound Healing Tool for Image J (NIH, United States).

### Network Formation Assay

MSC network formation capacity was assessed by seeding WT or TNFR2 KO-MSCs (10^5^ cells/well) on phenol red-free Matrigel^TM^ (BD Biosciences) in 24-well plates for a duration of 24 h, at 37°C in 5% CO_2_. Cells were cultured in complete DMEM or EGM-2MV medium, Endothelial SingleQuots^TM^ Kit (Lonza).

Pictures were taken every 2 h, till 24 h, using a camera (Nikon D3100, Japan) installed on an inverted microscope (Nikon ECLIPSE T5100) with 4× and 10× objectives in phase-contrast mode.

For evaluating the impact of MSCs on HUVEC angiogenesis, 5 × 10^5^ WT or TNFR2 KO-MSCs were seeded in 75 cm^2^ flasks and cultured for 48 h, in EBM2 medium containing 5% FBS (Lonza) and 1% P/S/N (Gibco). After 2 days, conditioning media (CM) from both WT and TNFR2 KO-MSC cultures were taken and filtered through a 0.45 μm filter to eliminate the remaining MSCs and were added to HUVECs (P5). HUVECs cultured in EBM2 basal endothelial medium (Lonza) or EGM-2MV complete endothelial medium (Lonza) were used as negative and positive controls, respectively. Then, we analyzed the images for evaluating the tube length and the network structural complexity (number of branches, number of sides, ability to construct closed networks, and tube length) by means of the angiogenesis analyzer that is developed for the Image J software as previously described (Chevalier et al., 2014).

### Statistical Analysis

Prism (GraphPad) software was utilized for statistical analysis. To assess the normal distribution of data, the Shapiro–Wilk normality test was performed. Thereafter, depending on the number of comparatives, Student’s *t*-test or one-way ANOVA with *post hoc* analysis was used. Regarding cytometry analysis, we have normalized the mean fluorescence intensity (MFI) values with WT-MSC or T cell alone groups depending on the experimental conditions. This was followed by unpaired, two-tailed Student’s *t*-test, or one-way ANOVA for *p*-value generation. Data are presented as mean value ± SEM. ^∗^*P* < 0.05, ^∗∗^*P* < 0.01, ^∗∗∗^*P* < 0.001, ^****^P < 0.0001.

## Results

### Mesenchymal Stem Cell Characterization and Differentiation Capacity

**FIGURE 1:**
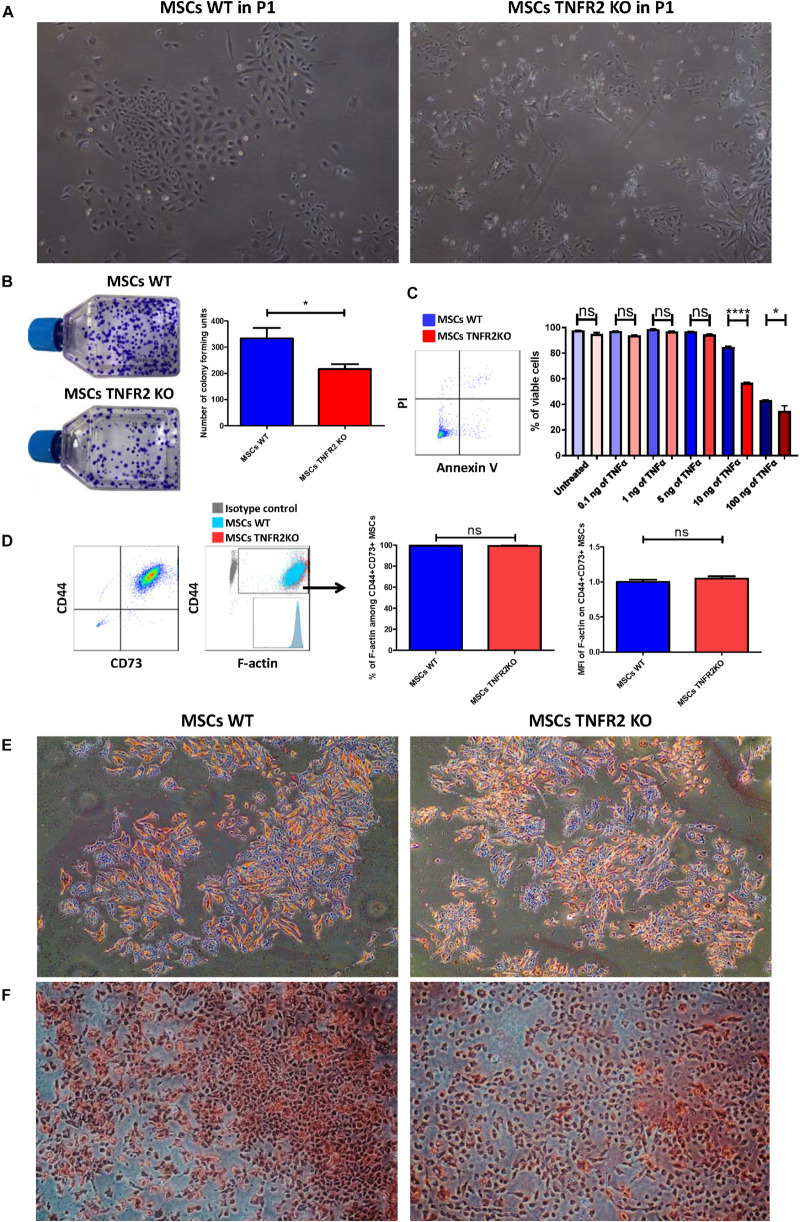
Mesenchymal stem cell (MSC) characterization and differentiation capacity. **(A)** Wild-type (WT)-MSCs in P1 demonstrate regular fibroblast-like form with spindle-shaped morphology (4×), while a more heterogeneous appearance with smaller cytoplasm is observed by their TNFR2 knockout (KO) counterparts in P1 (4×). **(B)** Colony-forming unit (CFU) assay reveals higher efficiency of WT-MSCs to form colony units in comparison with TNFR2 KO-MSCs. Results are collected from two independent experiments (*n* = 14). **(C)** Flow cytometric analysis of MSC viability assessment reveal the equivalent percentage of viable cells among untreated MSCs. Upon TNFα treatment for a duration of 24 h, WT-MSCs show more viability (annexin V^–^ PI^–^ population) in a dose-dependent manner. Cells were gated on total MSCs’ population (*n* = 6). **(D)** Flow cytometric analysis of the F-actin expression in WT and TNFR2 KO-MSCs (P3). Cells were gated on CD44^+^CD73^+^ MSCs. Mean fluorescence intensity (MFI) values have been normalized with WT-MSC group. Results are collected from two independent experiments (*n* = 6). **(E)** To evaluate adipogenic differentiation capacity, both MSC types (P3) were incubated in proper adipogenic differentiation medium for a duration of 3 weeks and then stained with Alizarin red S (4×). **(F)** To evaluate osteogenic differentiation, both MSC types (P3) were incubated in proper osteogenic differentiation medium for a duration of 17 days and then stained with Oil-Red-O (4×).

We have recently reported that BM-MSCs harvested from WT and TNFR2 KO mice have both normal physiological capabilities such as adherence to plastic plates and differentiation toward adipocytes and osteocytes (Beldi et al., 2020). Nevertheless, clear morphological differences were noticed notably in their early passages. TNFR2 KO-MSCs were more heterogeneous with considerably smaller cytoplasm. Moreover, they had less number of cells in each initial colony compared with WT-MSCs (Figure 1A). Assessing the ability of MSCs to form CFUs in passage 1 revealed that WT-MSCs formed significantly more colonies than TNFR2 KO-MSCs (Figure 1B). This was in accordance with their lower proliferation rate at P1, P2, and P3 (Supplementary Figure 1A). In this case, CPDT was longer for TNFR2 KO-MSCs compared with WT-MSCs in P1 (27.04 ± 0.27 h, versus 23.35 ± 0.04 h, respectively) and in P2 (27.17 ± 0.59 h versus 22.39 ± 0.08 h, respectively) (Supplementary Figure 1B). The TNFR2 KO-MSCs’ CPDT reached that of WT-MSCs in passage 3 (13.37 ± 0.01 h vs. 12.46 ± 0.01 h, respectively) (Supplementary Figure 1B). In order to evaluate the cell viability in the early passage (P1) after isolation, we have measured the expression of annexin V and PI among both MSC types. Our results demonstrated a high percentage of viable cells in untreated basal conditions among both WT and TNFR2 KO-MSCs with 97.76 and 95.06% of viability, respectively (Figure 1C). In addition, to explore the impact of the TNFα–TNFR2 axis on MSC viability we have treated them with five increasing doses of TNFα (i.e., 0.1, 1, 5, 10, and 100 ng/ml) for a duration of 24 h. We did not notice any difference in their viability until 5 ng/ml of TNFα treatment; however, in 10 and 100 ng/ml conditions, WT-MSCs were significantly more viable than their TNFR2 KO counterparts (Figure 1C).

Observing the morphological differences, we investigated whether there is a difference in their cytoplasmic microfilament constitution. Therefore, we measured filamentous-actin expression (F-actin) and surprisingly observed no difference in neither the percentage of expression nor the MFI of this protein (Figure 1D). We then explored if the absence of TNFR2 could change MSC differentiation capacity. While we did not notice a significant dissimilarity between their adipogenic differentiation potential (Figure 1E), we found a remarkable reduction in TNFR2 KO-MSC osteogenic capacity (Figure 1F).

### The Expression of Mesenchymal Stem Cell Characteristic Markers Is Diminished in the Absence of TNFR2

**FIGURE 2:**
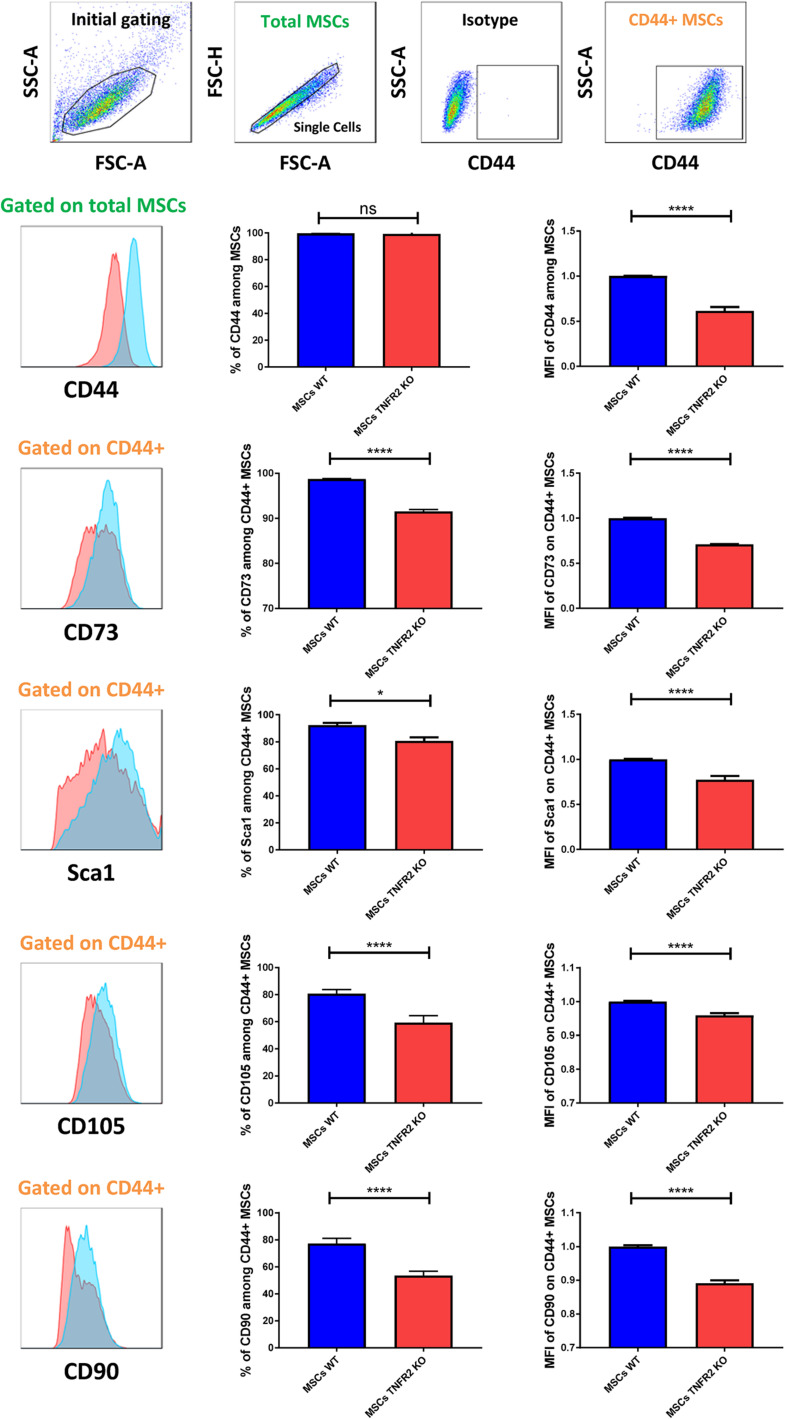
Mesenchymal stem cell (MSC) characteristic markers’ expression is diminished in the absence of TNFR2. This figure illustrates the surface expression of mouse MSC markers such as CD44, CD73, stem cell antigen-1 (Sca1), CD105, and CD90 in both MSC types (P3). To investigate the expression level of CD44 marker, cells were pre-gated on total MSCs (represented in green color). For the rest of the markers, cells were pre-gated on CD44^+^ MSCs (represented in orange color). Mean fluorescence intensity (MFI) values have been normalized with wild-type (WT)-MSC group. Red histograms depict TNFR2 knockout (KO)-MSCs, and blue histograms depict WT-MSCs. The results of CD44 (*n* = 25) and Sca1 (*n* = 19) expression are collected from four different experiments. The results of CD73, CD105, and CD90 markers (*n* = 13) are collected from three independent experiments.

Our previous studies revealed that both WT and TNFR2 KO-MSCs express mouse MSC characteristic markers like CD44, Sca1, CD73, CD105, and CD90 and do not express CD45 common leukocyte and CD34 hematopoietic markers (Beldi et al., 2020). Accordingly, here we demonstrate that both BM-MSCs were able to express those markers (Figure 2). However, our further experiments to compare the expression level of each MSC characteristic marker showed that except for the percentage of CD44, the percentage and MFI of all other cytoplasmic markers were significantly diminished in TNFR2 KO-MSCs in comparison with WT-MSCs (Figure 2). Moreover, we did not see any CD45 or CD34 expression in neither of the MSCs (Supplementary Figure 2).

### TNFR2 Expression Modulates Mesenchymal Stem Cell Capacity to Produce Anti- and Pro-inflammatory Cytokines

**FIGURE 3:**
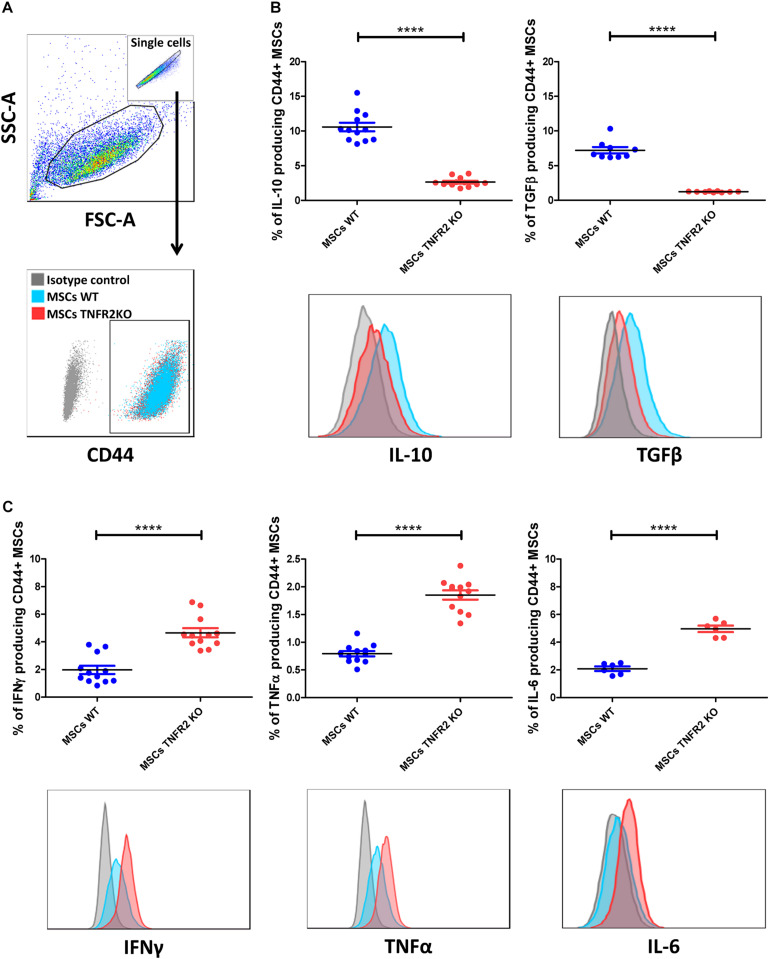
The TNFR2 expression modulates mesenchymal stem cell (MSC) capacity to produce anti-inflammatory and pro-inflammatory cytokines. Wild-type (WT) and TNFR2 knockout (KO)-MSCs were first activated by the addition of 10 ng/ml of TNFα. After 48 h, they were analyzed for their capacity to produce different anti-inflammatory and pro-inflammatory cytokines. **(A)** These flow cytometric representatives depict the initial gating strategy. Single cells were initially selected in SSC-A and FSC-A panels. Then, CD44^+^ MSCs were selected for further analysis. **(B)** Intracellular anti-inflammatory cytokine and **(C)** pro-inflammatory cytokine production was determined in WT and TNFR2 KO-MSCs. Gray histograms depict isotype controls, red histograms depict TNFR2 KO-MSCs, and blue histograms depict WT-MSCs. Each measured value is presented by a dot, and horizontal lines depict mean value ± SEM. Data are representative of three independent experiments. *n* = 12 for all cytokines except for IL-6 (*n* = 6) and TGFβ (*n* = 9).

Afterward, we tested whether blocking the TNFα–TNFR2 signaling pathway impacts MSC cytokine production pattern. WT and TNFR2 KO-MSCs were first activated by the addition of 10 ng/ml of TNFα. After 48 h, their capacity to produce different anti-inflammatory and pro-inflammatory cytokines was analyzed. Cells were initially gated on CD44, since this marker was merely equally expressed on both MSC types (Figure 3A). We first investigated the capacity of MSCs to produce IL-10 and TGFβ anti-inflammatory cytokines. Interestingly, we observed a dramatic decrease in the capacity of TNFR2 KO-MSCs to produce both cytokines (Figure 3B). Contrariwise, hampering the TNFα–TNFR2 axis led to a significant increase in IFNγ, TNFα, and IL-6 production by TNFR2 KO-MSCs compared with their WT counterparts (Figure 3C).

### Expression of TNFR2 by Mesenchymal Stem Cells Is Associated With Their Higher Nitric Oxide Production

**FIGURE 4:**
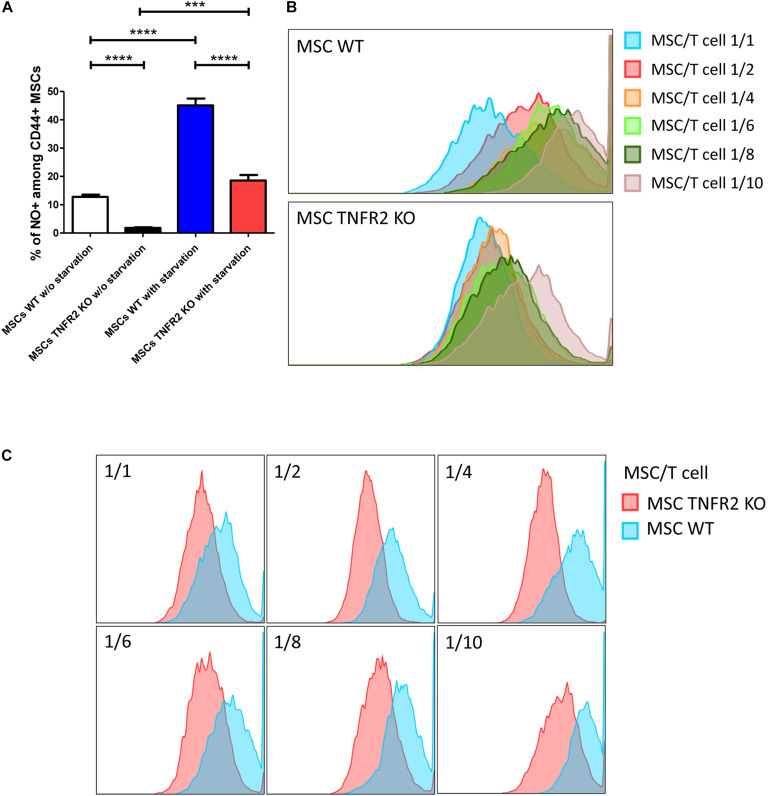
The expression of TNFR2 by mesenchymal stem cells (MSCs) is correlated to their higher NO production. NO production was evaluated by flow cytometric measurement. **(A)** Percentages of NO producing MSCs were identified at basal level without starvation (P2 and P3) and when cultured in medium containing 0.5% fetal bovine serum (FBS) creating cellular stress (P2 and P3). The first two bars represents the wild-type (WT) and TNFR2 knockout (KO)-MSCs cultured without starvation (*n* = 9), while the third and fourth bars are WT and TNFR2 KO-MSCs, respectively, cultured in starvation condition (*n* = 9). **(B)** To assess MSCs’ reaction in the presence of T cells, we have created an inflammatory condition by co-culturing both MSCs (P2 and P3) with six increasing ratios of activated T cells (1/1 up to 1/10 MSC/T cell ratio). **(C)** Comparing MSCs in each T cell dose revealed that the blockade of TNFR2 resulted in less NO production in every single ratio. Results are collected from three independent experiments.

One of the main mediators of murine MSC immunosuppressive and immunomodulatory effect is the NO production (Ren et al., 2008). Therefore, we investigated if there is a liaison between the TNFR2 expression and NO production by MSCs. It was already evidenced that MSCs do not secrete elevated amounts of NO in non-stressed conditions (Rahmat et al., 2013). Accordingly, we identified limited percentages of NO producing MSCs at basal level; however, even in this condition, WT-MSCs were more capable of NO production (Figure 4A). In order to create cellular stress, we have starved MSCs by growing them in a medium containing 0.5% FBS. This condition effectively enhanced NO production by both WT-MSCs (from 12.7% before starvation to 45.1% after starvation) and in TNFR2 KO-MSCs (from 1.8% before starvation to 18.5% after starvation). Due to the absence of TNFR2 pro-survival factor, in addition to the absence of the indispensable mediators present in FBS, starvation might have caused more cellular stress leading to a more effective increase in NO production by TNFR2 KO-MSCs; nevertheless, a more statistically significant NO production was observed by WT-MSCs (Figure 4A). We have recently demonstrated that TNFR2 KO-MSCs had a hampered ability to suppress T cells. In order to evaluate how MSCs react in the presence of T cells, we have created an inflammatory condition by co-culturing both MSCs with six increasing ratios of activated T cells (1/1 up to 1/10 MSC/T cell ratio). Our data showed that although both MSCs were able to produce more NO with regards to increasing T cell numbers, this effect was substantially more enhanced for WT-MSCs than TNFR2 KO-MSCs (Figure 4B). Comparing MSCs in each T cell dose revealed that the blockade of TNFR2 resulted in less NO production in every single ratio (Figure 4C).

### TNFR2 Expression by Mesenchymal Stem Cells Results in the Induction of More Immunosuppressive Tregs

**FIGURE 5:**
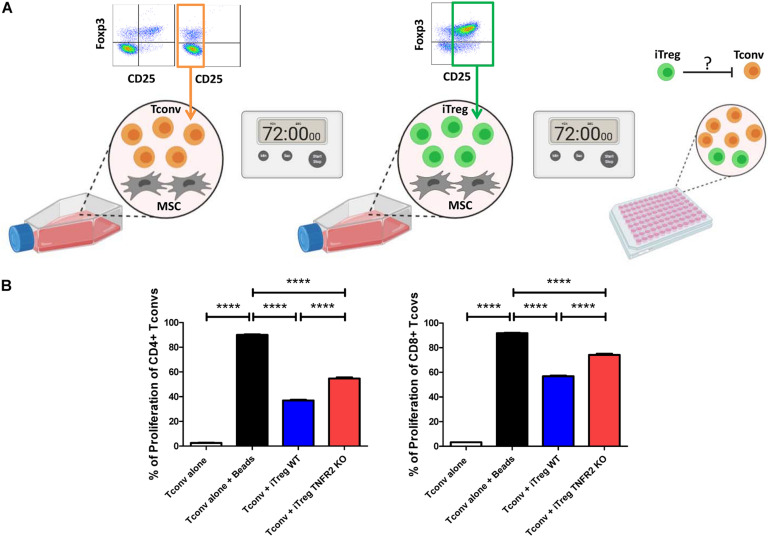
The TNFR2 Expression by mesenchymal stem cells (MSCs) is associated with the induction of Tregs with more immunosuppressive effect. **(A)** T cells were freshly isolated and depleted from CD25 subpopulation in order to eliminate natural Tregs and highly activated T cells. CD3^+^CD25^–^ Tconvs (orange population) were then added to wild-type (WT) and TNFR2 knockout (KO)-MSCs in a fixed 1/10 MSC/T cell ratio. After 72 h, CD4^+^CD25^+^Foxp3^+^ induced regulatory T cells (iTregs) generated in those co-cultures (green population) were put in an mixed lymphocyte reaction (MLR) test with newly isolated and activated mouse CFSE^+^CD3^+^CD25^–^ Tconvs in a fixed 1/5 iTreg/Tconv ratio. Then, the CD4^+^ and CD8^+^ proliferation capacity was measured by fluorescence-activated cell sorting (FACS). **(B)** Percentage of proliferation of CD4^+^ and CD8^+^ Tconvs in the presence of MSC induced Foxp3^+^ Tregs. Control groups consist of unstimulated T cells alone as depicted by the white columns (*n* = 6), while the stimulated T cells alone are depicted by the black columns (*n* = 6). The blue columns represent the stimulated T cells co-cultured with iTregs derived from WT-MSCs (*n* = 6), and the red columns represent the stimulated T cells co-cultured with iTregs derived from TNFR2 KO-MSCs (*n* = 6). Results are collected from two independent experiments. The graphical images were created with BioRender.com.

We have already depicted that the expression of TNFR2 is directly related to MSC ability to induce CD4^+^CD25^+^Foxp3^+^ and CD8^+^CD25^+^Foxp3^+^ Tregs (Beldi et al., 2020). Since TNFR2 KO-MSCs had less IL-10, TGFβ, and NO production rate, we have assessed if iTregs derived from these cells display less immunosuppressive effect. Therefore, T cells were freshly isolated and depleted from the CD25 subpopulation. This step was performed to eliminate natural Tregs and highly activated T cell populations. CD3^+^CD25^–^ Tconvs were then co-cultured with WT and TNFR2 KO-MSCs in 1/10 MSC/T cell ratio. After 72 h, CD4^+^CD25^+^Foxp3^+^ iTregs generated in those co-cultures were evaluated with an MLR test with freshly isolated, CFSE-labeled activated mouse CD3^+^CD25^–^ Tconvs in a fixed 1/5 iTreg/Tconv ratio (Figure 5A). In this setting, iTregs derived from WT-MSCs were significantly more immunosuppressive against both CD4^+^ and CD8^+^ Tconvs than iTregs derived from TNFR2 KO-MSCs (Figure 5B). iTregs derived from WT-MSCs suppressed 58.91% of CD4 and 38.16% of CD8 T cell proliferation. These values were 39.19 and 19.19% of CD4 and CD8 T cell suppression, respectively, after co-culturing with TNFR2 KO-MSCs (Figure 5B).

### Expression of TNFR2 by Mesenchymal Stem Cells Is Related to Their Increased Wound Healing Property

**FIGURE 6:**
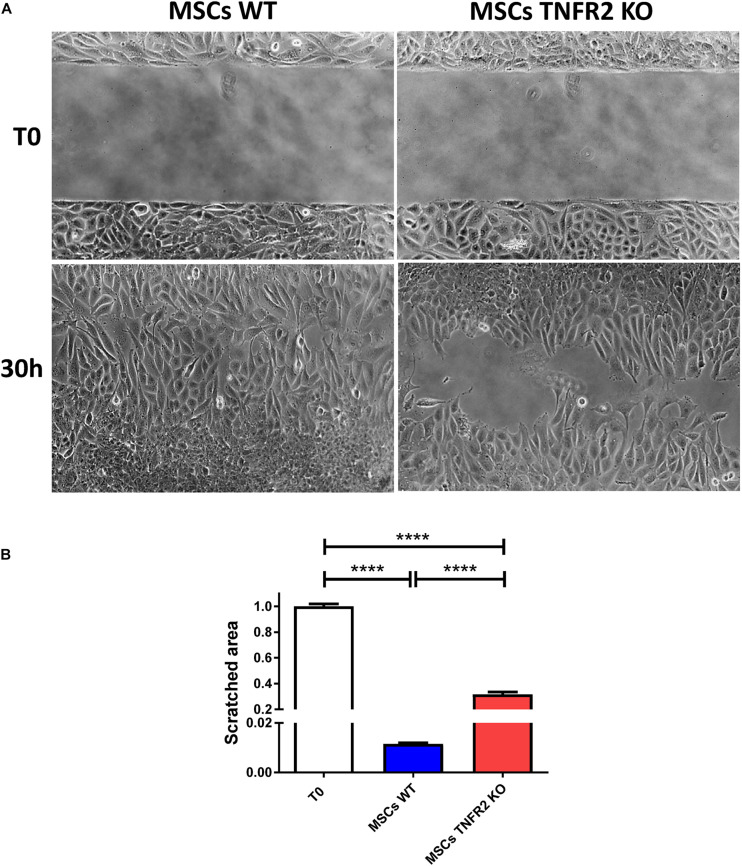
The TNFR2 expression by mesenchymal stem cells (MSCs) is associated with their appropriate wound healing property. The ability of wild-type (WT) and TNFR2 knockout (KO)-MSCs (P2 and P3) to close a scratch in the monolayer cell surface was measured. **(A)** Pictures were taken every 2 h, using objectives 4× and 10× of the microscope in phase-contrast mode to capture the wound area filling. **(B)** The size of the scratched area was measured in order to evaluate the wound area decrease. Results are collected from three independent experiments (*n* = 6). T0, time 0; and 30 h, 30 hours.

We then assessed the ability of MSCs to close a scratch wound in the monolayer cell surface. Our data revealed a much stronger wound healing capacity for WT-MSCs compared with TNFR2 KO-MSCs (Figure 6A). WT-MSCs achieved close to more than 99% of the scratched area within 30 h, while their TNFR2 KO counterparts reached only 69% within the same period of time (Figure 6B).

### Expression of TNFR2 by Mesenchymal Stem Cells Influences Their Tube Formation Property

**FIGURE 7:**
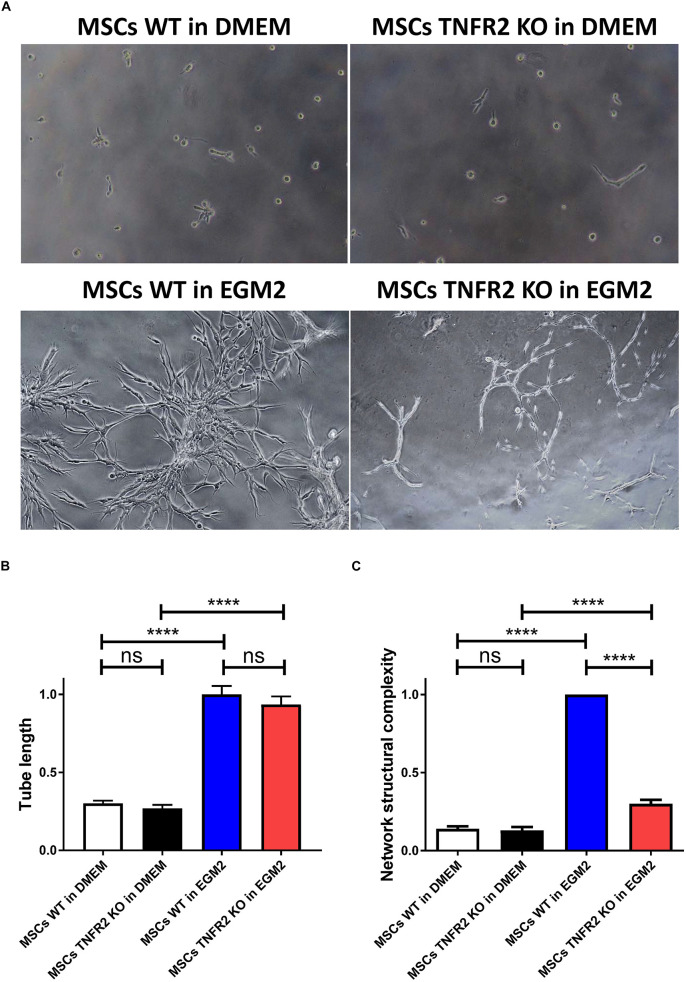
The TNFR2 expression by mesenchymal stem cells (MSCs) is associated with their enhanced tube formation property. To evaluate the involvement of TNFR2 in MSC regenerative feature, wild-type (WT) and TNFR2 knockout (KO)-MSCs (P2 and P3) were cultured on Matrigel using either Dulbecco’s modified Eagle’s medium (DMEM) standard medium or EGM2 endothelial medium. **(A)** Pictures were taken every 2 h, using objectives 4× and 10× of the inverted microscope in phase-contrast mode. **(B)** The tube length and **(C)** network structural complexity of WT and TNFR2 KO-MSCs were further evaluated. Results are collected from three independent experiments (*n* = 10).

One of the main characteristics of MSCs is their capability to exert pro-angiogenic functions through the construction of tubular complex structures in the presence of proper angiogenic and extracellular matrix mediators (Shen et al., 2015; Yu et al., 2018). To evaluate the involvement of TNFR2 in this regenerative feature, WT and TNFR2 KO-MSCs were cultured on Matrigel using either DMEM standard medium or EC growth medium (EGM2) containing a variety of pro-angiogenic factors such as VEGF, FGF, EGF, and IGF. As expected, we noticed that neither of the MSCs could form tubular complex structures in the absence of angiogenic factors (Figure 7A). On the contrary, while culturing WT-MSCs in EGM2 led to complex tubular and network formation, TNFR2 KO-MSCs were significantly less performant (Figure 7A). According to our acquired results, both MSCs constructed tubes with similar lengths (Figure 7B). However, comparing their network complexity revealed a much more developed, multibranchial, and dense 3D structures for WT-MSCs, which were some of the important features that were plainly deficient in TNFR2 KO-MSCs (Figure 7C).

### Expression of TNFR2 Is Crucial for Mesenchymal Stem Cells to Support Endothelial Cell Angiogenic Function

**FIGURE 8:**
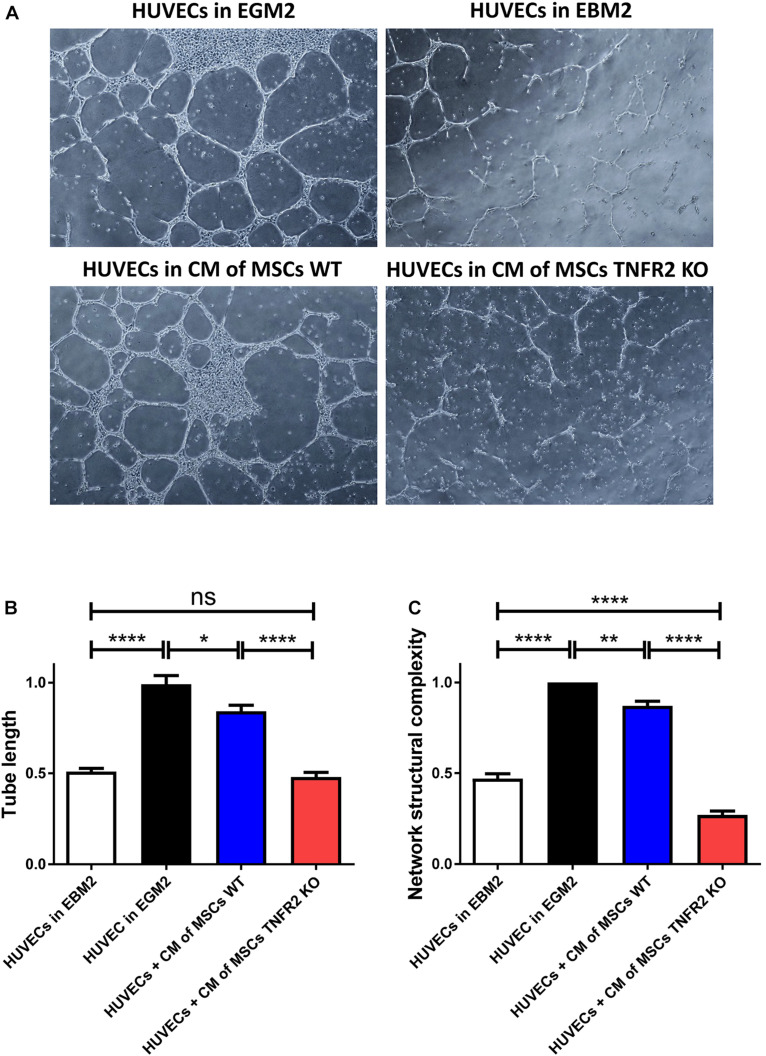
Expression of TNFR2 is crucial for mesenchymal stem cells (MSCs) to support endothelial cell (EC) angiogenic function. To evaluate the impact of MSCs on human umbilical vein endothelial cell (HUVEC) angiogenic capacity, wild-type (WT) or TNFR2 knockout (KO)-MSCs (P2 and P3) were cultured in complete Dulbecco’s modified Eagle’s medium (DMEM) medium. After 2 days, CM were taken, filtered, and added to HUVECs on Matrigel. HUVECs cultured in EBM2 basal medium were used as negative control, and HUVECs cultured in EGM2 complete medium were used as positive control. **(A)** Pictures were taken every 2 h, using objectives 4× and 10× of the inverted microscope in phase-contrast mode. Images were further analyzed to evaluate **(B)** the tube length and **(C)** the network structural complexity. Results are collected from three independent experiments (*n* = 10). CM, conditioned media.

MSCs have been shown to have supportive and protective effects toward EC angiogenic function mostly through secretion of pro-angiogenic factors like VEGF, TGFβ, and HGF (Watt et al., 2013; Beckermann et al., 2008; Batlle et al., 2019; Maacha et al., 2020). Here, we aimed to investigate whether there exists a relation between the TNFR2 expression and the MSC pro-angiogenic function. HUVECs were selected as the responsive cells to assess the tube formation function on Matrigel. Although HUVECs were unable to form tubular networks in endothelial basal medium (EBM2), the addition of pro-angiogenic factors (EGM2 medium) led to the formation of complex tubular structures (Figure 8A). We then evaluated the impact of CM of MSCs on HUVEC tube formation. WT and TNFR2 KO-MSCs were cultured in EBM2 medium; and after 48 h, their CM were collected and added to HUVECs. Surprisingly, while we witnessed a complete pro-angiogenic effect of CM from WT-MSCs, this effect was considerably impaired with CM of TNFR2 KO-MSCs (Figure 8A). HUVECs in CM of WT-MSCs formed significantly longer tubes (Figure 8B) and much more complex and closed network structures (Figure 8C).

## Discussion

The combination of immunomodulatory and regenerative properties of MSCs made these cells the most frequently investigated stem cells for clinical applications during the last couple of decades. This led to a large number of publications describing their therapeutic potential in a variety of disorders, often by suggesting new mechanisms of action from genomic to proteomic investigations. MSCs have been demonstrated to react unexpectedly in different inflammatory conditions. Endeavors to discover MSC behavior in presence of inflammatory cytokines such as TNFα surprisingly revealed enhanced immunosuppressive and regenerative effects in a compensatory feedback (English et al., 2007; Szabó et al., 2015; Broekman et al., 2016; Yang H.-M. et al., 2018; Noronha et al., 2019). As already mentioned in the *Introduction*, TNFα recognizes two transmembrane receptors (TNFR1 and TNFR2), which distinctly initiate downstream signaling that terminates in biological fates from one extreme to the other (Faustman and Davis, 2013; Wajant and Siegmund, 2019). TNFR1 is omnipresent on all cells, while TNFR2 is limitedly expressed on few cells including MSCs. Therefore, understanding the exact role of TNFR2 and the reason for its expression seems crucial.

Our previous attempts to unravel the role of TNFα–TNFR2 signaling pathway in other cell types led to the conclusion that this axis controls principal immunoregulatory and protective functions. For instance, it has been shown that TNFR2 controls Treg, Breg, MDSC, and EPC immunosuppressive effect (Polz et al., 2014; Leclerc et al., 2016; Ticha et al., 2017; Naserian et al., 2020). Interestingly, TNFα pre-treatment resulted in increased Treg and MDSC immunosuppressive property (Hu et al., 2014; Pierini et al., 2016). In case of MSCs, signaling through TNFR2 was shown to support regenerative functions and to be essential for their therapeutic effect in treating inflammatory and autoimmune disorders (Kelly et al., 2010; Yan et al., 2018).

In this study, we wanted to understand more precisely the exact role of TNFR2 in MSCs. To this goal, we first compared the expression of MSC specific markers on BM-MSCs harvested from WT and TNFR2 KO mice. Our data showed that TNFR2 KO-MSCs had lower expression of CD44, Sca1, CD105, CD73, and CD90 principal markers. Despite these phenotypical differences, due to the absence of a specific marker, it is difficult to determine whether TNFR2 KO-MSCs have already committed toward another lineage. However, as supported by other research publications and the results of our study, we think that the reduction in the TNFR2 KO-MSC characterization markers is associated with less functionality linked to each or a combination of those markers rather than a conversion toward another lineage. For example, the expression of CD90 was mostly associated with MSC immunosuppressive effect (Campioni et al., 2009). Accordingly, we reported that mouse TNFR2 KO-MSCs have significantly lower immunosuppressive and immunomodulatory effect against T cells (Beldi et al., 2020). CD73 marker was associated with MSC reparative and regenerative properties. The injection of CD73^low^ MSCs into mouse heart was shown to be much less effective to repair myocardial infarction than CD73^high^ MSCs (Tan et al., 2019). In agreement, TNFR2 expression was shown to be crucial in MSC cardiac protection following acute ischemia (Kelly et al., 2010). CD105, CD73, and CD90 markers were also associated with MSC differentiation capacity (Arufe et al., 2010; Vaculik et al., 2012; Lv et al., 2014). Our results demonstrated that although we did not observe a clear difference in MSCs’ adipogenic differentiation, the absence of TNFR2 caused a significant decrease in MSCs’ osteogenic differentiation capacity. It would be interesting to further assess the role of TNFR2 in MSC differentiation capacity by studying the expression of different proteins and their genes such as PPARγ, BMPR1, and FABP4 that are shown to be upregulated in adipogenesis (Farmer, 2005; Moseti et al., 2016; Liu et al., 2019) and RUNX2 that is related to MSC osteogenesis (Thiagarajan et al., 2017). Our investigation on chondrogenic differentiation revealed a disrupted capacity for TNFR2 KO-MSCs (data not shown). Further studies are indeed necessary to confirm these preliminary results.

Our previous research works correlated the expression of TNFR2 to MSC immunosuppressive effect (Beldi et al., 2020). Besides, TNFα priming was shown to increase IL-10 and TGFβ secretion by MSCs (Putra et al., 2018). This encouraged us to evaluate the TNFα–TNFR2 involvement in the production of MSC immunomodulatory mediators like cytokines and NO. TNFR2 blockade led to increased levels of IFNγ, TNFα, and IL-6 pro-inflammatory and decreased IL-10 and TGFβ anti-inflammatory cytokines and NO production. This is in accordance with other studies showing a direct correlation between the expression of TNFR2 and enhanced IL-10, TGFβ, and HLA-G secretion by EPCs (Naserian et al., 2020) and Bregs (Ticha et al., 2017).

The secretion of anti-inflammatory mediators by MSCs is one of the main mechanisms mediating their Treg induction capacity. We have recently reported that in comparison with WT-MSCs, TNFR2 KO-MSCs were considerably less able to induce CD4^+^ and CD8^+^ Tregs (Beldi et al., 2020). Here, we complete our preceding work by demonstrating that TNFR2 deficiency leads to the induction of Tregs with remarkably less immunosuppressive effect. This piece of information is specifically interesting since it highlights the importance of TNFR2 not only in MSC immunosuppressive effect but also in other cells that are in contact with them such as T cells. It seems essential to evaluate the role of TNFR2 on other cells such as tolerogenic dendritic cells (tDCs) and M2 macrophages that could be also induced from MSCs.

In the second part of this work, we aimed to investigate the involvement of TNFR2 in some principal MSC regenerative properties including wound healing, tube formation, and pro-angiogenic effects. We demonstrated that hampering TNFR2 molecule will cause diminished MSC wound healing and tube formation capacities.

MSC involvement in angiogenesis has been already proven. By a direct cellular contribution to vascular system, they can induce a protective vascular function. Rapidly after their injection, MSCs induce primal immature vascular tubes linked to the host circulation. Surprisingly, this phenomenon was shown to be even faster than host EC and smooth muscle cell (SMC) recruitment to neovascularization site (Dufourcq et al., 2008). The angiogenic efficiency of MSCs was reported to be remarkably variant depending on their originated source, resulting in heterogeneous regenerative outcomes (Xu et al., 2017; Lu et al., 2018). Our data reveal TNFR2 as key regulator of MSC angiogenic properties. This point is interesting since enriching TNFR2^+^ MSCs could potentially provide MSCs with the most homogeneous and highest pro-angiogenic functions. Blocking TNFR2 by an appropriate antagonist, on the other hand, could efficiently hamper the MSC angiogenic and immunoregulatory functions. This is specifically crucial in cancer treatment where MSCs have been shown to be strongly involved in favoring tumor growth by supporting angiogenic and immunosuppressive microenvironment mostly via TGFβ-dependent mechanism (Zhang T. et al., 2013; Li et al., 2016; Trivanović et al., 2016; Yang et al., 2017; Batlle et al., 2019; Lee et al., 2019), a cytokine that we showed to be significantly less produced by TNFR2 KO-MSCs.

MSCs can also favor angiogenesis through supporting EC function via promoting the secretion of pro-angiogenic factors (Fransson et al., 2015; Premer et al., 2015). Here, by evaluating the impact of CM of MSCs on HUVEC angiogenic capacity, we demonstrated that in complete opposite to WT-MSCs, CM form TNFR2 KO-MSCs that cannot support HUVEC tube formation. This is in accordance with other studies showing that the therapeutic effect of MSCs is through paracrine mechanism, which is strictly regulated by Rap1/NF-κB signaling pathway (Zhang Y. et al., 2013; Zhang et al., 2015). Interestingly, the TNFα–TNFR2 interaction can activate NF-κB signaling pathway through TNF receptor-associated factor (TRAF) involvement (Faustman and Davis, 2013). Indeed, previous studies have reported that the VEGFR2 signaling pathway is defective in TNFR2 KO mice (Luo et al., 2006). Additionally, interfering in the TNFR2 signaling pathway impacts the secretion of pro-angiogenic factors such as HGF, IGF-1, and VEGF by MSC (Wang et al., 2006; Crisostomo et al., 2008; Zhang et al., 2010; Yan et al., 2018). The same phenomenon was observed in other cells such as EPCs linking the TNFR2 expression to VEGF, IGF, HGF, and IL-8 production and consequently to their angiogenic function (Yoshida et al., 1997; Hoefer et al., 2002; Goukassian et al., 2007). The principal novelty of our work is that we have measured some other mediators that are as crucial as classical growth factors in angiogenesis such as NO and TGFβ. NO has a central role in starting and stimulating angiogenesis through a direct effect and via induction of VEGF and FGF (Kang et al., 2020; Yamamoto et al., 2020). To our knowledge, this is the first report that correlates TNFR2 expression by MSCs to their NO production capacity.

In this study, we have used MSCs harvested from the BM, which is considered as an adult source for these cells. Several disadvantages have been attributed to adult tissue sources of MSCs including the heterogeneity, batch to batch variations, cellular senescence, and limited proliferative potency (Lukomska et al., 2019; Musiał-Wysocka et al., 2019). MSCs can be also derived from induced pluripotent stem cells (iPSCs). It has been demonstrated that human iPSC-derived MSCs (iPSC-MSCs) possess higher proliferative and immunomodulatory potentials (Zhang et al., 2012; Bloor et al., 2020). Besides, due to the higher expression of TNFαIP2, iPSC-MSCs are more responsive to TNFα-induced tunneling nanotube formation for mitochondrial transfer and have been shown to be more protective against cardiomyocyte damage (Zhang et al., 2016). Thus, it is worthy to assess the variation of expression rate and the exact implication of TNFR2 molecule in these MSC sources as well. Furthermore, it would be interesting to demonstrate if restoration of the TNFR2 expression in TNFR2 deficient MSCs can rescue their impaired regenerative and immunological functions. In the end, blocking the TNFR1 signaling pathway, sorting the TNFR2^+^ MSCs, and upregulating the TNFR2 molecule via its specific agonist to assess the potentially increased MSC biological function are among the missing information in this current work. Altogether, this work supports the strong implication of the TNFR2 expression in MSC biological and functional properties, especially in their immunological and regenerative potentials. We strongly believe that this study paves the way for further *in vitro* and *in vivo* explorations in this field.

## Data Availability Statement

The raw data supporting the conclusions of this article will be made available by the authors, without undue reservation.

## Ethics Statement

All experimental procedures were performed in accordance with the European Community Council Directive (2010/63/UE) for the care and use of laboratory animals. Experimental protocols were approved by the local ethics committee “Comité d’éthique en expérimentation animal Charles Darwin No 5” under the number 02811.03 in compliance with European Union guidelines.

## Author Contributions

SN and GU conceived the study. GB, SB, CL, MNB, and SN performed the experiments. GB, SB, and SN analyzed the data. GB and SN wrote the manuscript. MEA, GU, and SN reviewed the manuscript. SN and GU revised the manuscript. All authors contributed to the article and approved the submitted version.

## Conflict of Interest

SN is the CEO of CellMedEx Company. The remaining authors declare that the research was conducted in the absence of any commercial or financial relationships that could be construed as a potential conflict of interest.

## Abbreviations

BM-MSCs: bone marrow-derived mesenchymal stem cells
CD: cluster of differentiation
CPDT: cell population doubling time
CFSE: carboxyfluorescein succinimidyl ester
CFUs: colony-forming units
CM: conditioning media
EPCs: endothelial progenitor cells
HGF: hepatocyte growth factor
IFNγ: interferon gamma
IGF-1: insulin-like growth factor 1
IL: interleukin
iPSCs: induced pluripotent stem cells
iTreg: induced regulatory T cell
MACS: magnetic-activated cell sorting
MDCSs: myeloid-derived suppressive cells
MFI: mean fluorescence intensity
MSCs: mesenchymal stem cells
P: passage
Sca1: stem cells antigen-1
Tconv: conventional T cell
TNFR1: tumor necrosis factor receptor 1
TNFR2 KO: tumor necrosis factor receptor 2 knockout
TNFR2: tumor necrosis factor receptor 2
TNFα: tumor necrosis factor alpha
Treg: Regulatory T cell
VEGF: vascular endothelial growth factor.

## References

[B1] Afshari A. Shamdani S. Uzan G. Naserian S. Azarpira N. (2020). Different approaches for transformation of mesenchymal stem cells into hepatocyte-like cells. *Stem Cell Res. Ther.* 11:54. 10.1186/s13287-020-1555-8 32033595 PMC7007672

[B2] Arufe M. C. De la Fuente A. Fuentes I. de Toro F. J. Blanco F. J. (2010). Chondrogenic potential of subpopulations of cells expressing mesenchymal stem cell markers derived from human synovial membranes. *J. Cell. Biochem.* 111 834–845. 10.1002/jcb.22768 20665538

[B3] Bai M. Zhang L. Fu B. Bai J. Zhang Y. Cai G. (2018). IL-17A improves the efficacy of mesenchymal stem cells in ischemic-reperfusion renal injury by increasing treg percentages by the COX-2/PGE2 pathway. *Kidney Int.* 93 814–825. 10.1016/j.kint.2017.08.030 29132705

[B4] Baldari S. Di Rocco G. Piccoli M. Pozzobon M. Muraca M. Toietta G. (2017). Challenges and strategies for improving the regenerative effects of mesenchymal stromal cell-based therapies. *Int. J. Mol. Sci.* 18:2087. 10.3390/ijms18102087 28974046 PMC5666769

[B5] Bao C. Guo J. Lin G. Hu M. Hu Z. (2008). TNFR Gene-Modified Mesenchymal Stem Cells Attenuate Inflammation and Cardiac Dysfunction Following MI. *Scand. Cardiovasc. J. SCJ* 42 56–62. 10.1080/14017430701543556 17852784

[B6] Bao C. Guo J. Zheng M. Chen Y. Lin G. Hu M. (2010). Enhancement of the survival of engrafted mesenchymal stem cells in the ischemic heart by TNFR gene transfection. *Biochem. Cell Biol. Biochim. Biol. Cell.* 88 629–634. 10.1139/O10-018 20651834

[B7] Batlle R. Andrés E. Gonzalez L. Llonch E. Igea A. Gutierrez-Prat N. (2019). Regulation of tumor angiogenesis and mesenchymal–endothelial transition by P38α through TGF-β and JNK signaling. *Nat. Commun.* 10:3071. 10.1038/s41467-019-10946-y 31296856 PMC6624205

[B8] Beckermann B. M. Kallifatidis G. Groth A. Frommhold D. Apel A. Mattern J. (2008). VEGF expression by mesenchymal stem cells contributes to angiogenesis in pancreatic carcinoma. *Br. J. Cancer* 99 622–631. 10.1038/sj.bjc.6604508 18665180 PMC2527820

[B9] Beldi G. Khosravi M. Abdelgawad M. E. Salomon B. L. Uzan G. Haouas H. (2020). TNFα/TNFR2 signaling pathway: an active immune checkpoint for mesenchymal stem cell immunoregulatory function. *Stem Cell Res. Ther.* 11:281. 10.1186/s13287-020-01740-5 PMC7364521 32669116

[B10] Bloor A. J. C. Patel A. Griffin J. E. Gilleece M. H. Radia R. Yeung D. T. (2020). Production, safety and efficacy of IPSC-derived mesenchymal stromal cells in acute steroid-resistant graft versus host disease: a Phase I, multicenter, open-label, dose-escalation study. *Nat. Med.* 26 1720–1725. 10.1038/s41591-020-1050-x 32929265

[B11] Broekman W. Amatngalim G. D. de Mooij-Eijk Y. Oostendorp J. Roelofs H. Taube C. (2016). TNF-α and IL-1β-activated human mesenchymal stromal cells increase airway epithelial wound healing in vitro via activation of the epidermal growth factor receptor. *Respir. Res.* 17:3. 10.1186/s12931-015-0316-1 26753875 PMC4710048

[B12] Campioni D. Rizzo R. Stignani M. Melchiorri L. Ferrari L. Moretti S. (2009). Decreased positivity for CD90 on human mesenchymal stromal cells (MSCs) is associated with a loss of immunosuppressive activity by MSCs. *Cytometry B Clin. Cytom.* 76 225–230. 10.1002/cyto.b.20461 18985728

[B13] Chen H. Min X.-H. Wang Q.-Y. Leung F. W. Shi L. Zhou Y. (2015). Pre-activation of mesenchymal stem cells with TNF-α, IL-1β and Nitric oxide enhances its paracrine effects on radiation-induced intestinal injury. *Sci. Rep.* 5:8718. 10.1038/srep08718 25732721 PMC4346809

[B14] Chen Z. Palmer T. D. (2013). Differential roles of TNFR1 and TNFR2 signaling in adult hippocampal neurogenesis. *Brain. Behav. Immun.* 30 45–53. 10.1016/j.bbi.2013.01.083 23402793 PMC3641155

[B15] Chevalier F. Lavergne M. Negroni E. Ferratge S. Carpentier G. Gilbert-Sirieix M. (2014). Glycosaminoglycan mimetic improves enrichment and cell functions of human endothelial progenitor cell colonies. *Stem Cell Res.* 12 703–715. 10.1016/j.scr.2014.03.001 24681520

[B16] Crisostomo P. R. Wang Y. Markel T. A. Wang M. Lahm T. Meldrum D. R. (2008). Human mesenchymal stem cells stimulated by TNF-Alpha, LPS, or hypoxia produce growth factors by an NF Kappa B- but Not JNK-dependent mechanism. *Am. J. Physiol. Cell Physiol.* 294 C675–C682. 10.1152/ajpcell.00437.2007 18234850

[B17] Daneshmandi S. Karimi M. H. Pourfathollah A. A. (2017). TGF-β engineered mesenchymal stem cells (TGF-β/MSCs) for treatment of Type 1 Diabetes (T1D) mice model. *Int. Immunopharmacol.* 44 191–196. 10.1016/j.intimp.2017.01.019 28110219

[B18] de Witte S. F. H. Franquesa M. Baan C. C. Hoogduijn M. J. (2015). Toward development of IMesenchymal stem cells for immunomodulatory therapy. *Front. Immunol.* 6:648. 10.3389/fimmu.2015.00648 26779185 PMC4701910

[B19] Dominici M. Le Blanc K. Mueller I. Slaper-Cortenbach I. Marini F. Krause D. (2006). Minimal criteria for defining multipotent mesenchymal stromal cells. The international society for cellular therapy position statement. *Cytotherapy* 8 315–317. 10.1080/14653240600855905 16923606

[B20] Doucet C. Ernou I. Zhang Y. Llense J. Begot L. Holy X. (2005). Platelet lysates promote mesenchymal stem cell expansion: a safety substitute for animal serum in cell-based therapy applications. *J. Cell Physiol.* 236 228–236. 10.1002/jcp.20391 15887229

[B21] Dufourcq P. Descamps B. Tojais N. F. Leroux L. Oses P. Daret D. (2008). Secreted frizzled-related Protein-1 enhances mesenchymal stem cell function in angiogenesis and contributes to neovessel maturation. *Stem Cells* 26 2991–3001. 10.1634/stemcells.2008-0372 18757297

[B22] English K. Barry F. P. Field-Corbett C. P. Mahon B. P. (2007). IFN-Gamma and TNF-Alpha differentially regulate immunomodulation by murine mesenchymal stem cells. *Immunol. Lett.* 110 91–100. 10.1016/j.imlet.2007.04.001 17507101

[B23] Farmer S. R. (2005). Regulation of PPARgamma activity during adipogenesis. *Int. J. Obes* 2005(29 Suppl. 1), S13–S16. 10.1038/sj.ijo.0802907 15711576

[B24] Faustman D. L. Davis M. (2013). TNF. receptor 2 and Disease: autoimmunity and regenerative medicine. *Front. Immunol.* 4:478. 10.3389/fimmu.2013.00478 24391650 PMC3870411

[B25] FrançoisM.Romieu-MourezR.LiM.GalipeauJ. (2012). Human MSC suppression correlates with cytokine induction of indoleamine 2,3-dioxygenase and bystander M2 macrophage differentiation. *Mol. Ther. J. Am. Soc. Gene Ther.* 20 187–195. 10.1038/mt.2011.189 21934657

[B26] Fransson M. Brännström J. Duprez I. Essand M. Le Blanc K. Korsgren O. (2015). Mesenchymal stromal cells support endothelial cell interactions in an intramuscular islet transplantation model. *Regen. Med. Res.* 3:1. 10.1186/s40340-015-0010-9 26430512 PMC4589952

[B27] Friedenstein A. J. Petrakova K. V. Kurolesova A. I. Frolova G. P. (1968). Heterotopic of bone marrow. analysis of precursor cells for osteogenic and hematopoietic tissues. *Transplantation* 6 230–247. 5654088

[B28] Goukassian D. A. Qin G. Dolan C. Murayama T. Silver M. Curry C. (2007). Tumor necrosis factor-alpha receptor P75 is required in ischemia-induced neovascularization. *Circulation* 115 752–762. 10.1161/CIRCULATIONAHA.106.647255 17261656

[B29] Han I. Kwon B.-S. Park H.-K. Kim K. S. (2017). Differentiation potential of mesenchymal stem cells is related to their intrinsic mechanical properties. *Int. Neurourol. J.* 21(Suppl. 1), S24–S31. 10.5213/inj.1734856.428 28446012 PMC5426435

[B30] Hoefer I. E. van Royen N. Rectenwald J. E. Bray E. J. Abouhamze Z. Moldawer L. L. (2002). Direct evidence for tumor necrosis factor-alpha signaling in arteriogenesis. *Circulation* 105 1639–1641. 10.1161/01.cir.0000014987.32865.8e 11940540

[B31] Hu C. Li L. (2018). Preconditioning influences mesenchymal stem cell properties in vitro and in vivo. *J. Cell. Mol. Med.* 22 1428–1442. 10.1111/jcmm.13492 29392844 PMC5824372

[B32] Hu X. Li B. Li X. Zhao X. Wan L. Lin G. (2014). Transmembrane TNF-α promotes suppressive activities of myeloid-derived suppressor cells via TNFR2. *J. Immunol.* 192 1320–1331. 10.4049/jimmunol.1203195 24379122

[B33] Huang C. Dai J. Zhang X. A. (2015). Environmental physical cues determine the lineage specification of mesenchymal stem cells. *Biochim. Biophys. Acta* 1850 1261–1266. 10.1016/j.bbagen.2015.02.011 25727396 PMC4411082

[B34] Kang M.-L. Kim H.-S. You J. Choi Y. S. Kwon B.-J. Park C. H. (2020). Hydrogel cross-linking-programmed release of nitric oxide regulates source-dependent angiogenic behaviors of human mesenchymal stem cell. *Sci. Adv.* 6:eaay5413. 10.1126/sciadv.aay5413 32133403 PMC7043909

[B35] Kelly M. L. Wang M. Crisostomo P. R. Abarbanell A. M. Herrmann J. L. Weil B. R. (2010). TNF Receptor 2, Not TNF Receptor 1, Enhances mesenchymal stem cell-mediated cardiac protection following acute ischemia. *Shock* 33 602–607. 10.1097/SHK.0b013e3181cc0913 19953003 PMC3076044

[B36] Khosravi M. Azarpira N. Shamdani S. Hojjat-Assari S. Naserian S. Karimi M. H. (2018a). Differentiation of umbilical cord derived mesenchymal stem cells to hepatocyte cells by transfection of MiR-106a. MiR-574-3p, and MiR-451. *Gene* 667 1–9. 10.1016/j.gene.2018.05.028 29763649

[B37] Khosravi M. Bidmeshkipour A. Cohen J. L. Moravej A. Hojjat-Assari S. Naserian S. (2018b). Induction of CD4+CD25+FOXP3+ Regulatory T Cells by mesenchymal stem cells is associated with modulation of ubiquitination factors and TSDR demethylation. *Stem Cell Res. Ther.* 9:273. 10.1186/s13287-018-0991-1 30359308 PMC6203284

[B38] Khosravi M. Bidmeshkipour A. Moravej A. Hojjat-Assari S. Naserian S. Karimi M. H. (2018c). Induction of CD4+CD25+Foxp3+ regulatory T Cells by mesenchymal stem cells is associated with RUNX Complex factors. *Immunol. Res.* 66 207–218. 10.1007/s12026-017-8973-4 29143918

[B39] Khosravi M. Karimi M. H. Hossein Aghdaie M. Kalani M. Naserian S. Bidmeshkipour A. (2017). Mesenchymal stem cells can induce regulatory T Cells via Modulating MiR-126a but Not MiR-10a. *Gene* 627 327–336. 10.1016/j.gene.2017.06.012 28600182

[B40] Leclerc M. Naserian S. Pilon C. Thiolat A. Martin G. H. Pouchy C. (2016). Control of GVHD by regulatory T cells depends on TNF produced by T Cells and TNFR2 expressed by regulatory T Cells. *Blood* 128 1651–1659. 10.1182/blood-2016-02-700849 27506541

[B41] Lee M. W. Ryu S. Kim D. S. Lee J. W. Sung K. W. Koo H. H. (2019). Mesenchymal stem cells in suppression or progression of hematologic malignancy: current status and challenges. *Leukemia* 33 597–611. 10.1038/s41375-018-0373-9 30705410 PMC6756083

[B42] Li G.-C. Zhang H.-W. Zhao Q.-C. Sun L. Yang J.-J. Hong L. (2016). Mesenchymal stem cells promote tumor angiogenesis via the action of transforming growth Factor B 1. *Oncol. Lett.* 11 1089–1094. 10.3892/ol.2015.3997 26893697 PMC4733964

[B43] Liu L. N. Wang G. Hendricks K. Lee K. Bohnlein E. Junker U. (2013). Comparison of drug and cell-based delivery: engineered adult mesenchymal stem cells expressing soluble tumor necrosis factor receptor ii prevent arthritis in mouse and rat animal models. *Stem Cells Transl. Med.* 2 362–375. 10.5966/sctm.2012-0135 23592838 PMC3667563

[B44] Liu Z. Wang P. Cen S. Gao L. Xie Z. Wu X. (2019). Increased BMPR1A expression enhances the adipogenic differentiation of mesenchymal stem cells in patients with ankylosing spondylitis. *Stem Cells Int.* 2019 4143167. 10.1155/2019/4143167 31827527 PMC6885782

[B45] Lu H. Wang F. Mei H. Wang S. Cheng L. (2018). Human adipose mesenchymal stem cells show more efficient angiogenesis promotion on endothelial colony-forming cells than umbilical cord and endometrium. *Stem Cells Int.* 2018:7537589. 10.1155/2018/7537589 30651736 PMC6311802

[B46] Lukomska B. Stanaszek L. Zuba-Surma E. Legosz P. Sarzynska S. Drela K. (2019). Challenges and controversies in human mesenchymal stem cell therapy. *Stem Cells Int.* 2019:9628536. 10.1155/2019/9628536 31093291 PMC6481040

[B47] Luo D. Luo Y. He Y. Zhang H. Zhang R. Li X. (2006). Differential functions of tumor necrosis Factor Receptor 1 and 2 Signaling in Ischemia-mediated arteriogenesis and angiogenesis. *Am. J. Pathol.* 169 1886–1898. 10.2353/ajpath.2006.060603 17071609 PMC1780200

[B48] Lv F.-J. Tuan R. S. Cheung K. M. C. Leung V. Y. L. (2014). Concise review: the surface markers and identity of human mesenchymal stem cells. *Stem Cells* 32 1408–1419. 10.1002/stem.1681 24578244

[B49] Maacha S. Sidahmed H. Jacob S. Gentilcore G. Calzone R. Grivel J.-C. (2020). Paracrine mechanisms of mesenchymal stromal cells in angiogenesis. *Stem Cells Int.* 2020:4356359. 10.1155/2020/4356359 32215017 PMC7085399

[B50] Maleki M. Ghanbarvand F. Reza Behvarz M. Ejtemaei M. Ghadirkhomi E. (2014). Comparison of mesenchymal stem cell markers in multiple human adult stem cells. *Int. J. Stem Cells* 7 118–126. 10.15283/ijsc.2014.7.2.118 25473449 PMC4249894

[B51] Moseti D. Regassa A. Kim W.-K. (2016). Molecular regulation of adipogenesis and potential anti-adipogenic bioactive molecules. *Int. J. Mol. Sci.* 17:124. 10.3390/ijms17010124 26797605 PMC4730365

[B52] Musiał-Wysocka A. Kot M. Majka M. (2019). The pros and cons of mesenchymal stem cell-based therapies. *Cell Transplant.* 28 801–812. 10.1177/0963689719837897 31018669 PMC6719501

[B53] Naserian S. Abdelgawad M. E. Afshar Bakshloo M. Ha G. Arouche N. Cohen J. L. (2020). The TNF/TNFR2 signaling pathway is a key regulatory factor in endothelial progenitor cell immunosuppressive effect. *Cell Commun. Signal.* 18:94. 10.1186/s12964-020-00564-3 32546175 PMC7298859

[B54] Nava M. M. Raimondi M. T. Pietrabissa R. (2012). Controlling self-renewal and differentiation of stem cells via mechanical cues. *J. Biomed. Biotechnol.* 2012:797410. 10.1155/2012/797410 23091358 PMC3471035

[B55] Noronha N. D. C. Mizukami A. Caliári-Oliveira C. Cominal J. G. Rocha J. L. M. Covas D. T. (2019). Priming approaches to improve the efficacy of mesenchymal stromal cell-based therapies. *Stem Cell Res. Ther.* 10:131. 10.1186/s13287-019-1224-y 31046833 PMC6498654

[B56] Park N. Rim Y. A. Jung H. Kim J. Yi H. Kim Y. (2017). Etanercept-synthesising mesenchymal stem cells efficiently ameliorate collagen-induced arthritis. *Sci. Rep.* 7:39593. 10.1038/srep39593 28084468 PMC5234034

[B57] Pierini A. Strober W. Moffett C. Baker J. Nishikii H. Alvarez M. (2016). TNF-α Priming enhances CD4+FoxP3+ Regulatory T-Cell suppressive function in murine GVHD prevention and treatment. *Blood* 128 866–871. 10.1182/blood-2016-04-711275 27365424 PMC4982455

[B58] Polz J. Remke A. Weber S. Schmidt D. Weber-Steffens D. Pietryga-Krieger A. (2014). Myeloid suppressor cells require membrane TNFR2 expression for suppressive activity. *Immun. Inflamm. Dis.* 2 121–130. 10.1002/iid3.19 25400932 PMC4217546

[B59] Pourgholaminejad A. Aghdami N. Baharvand H. Moazzeni S. M. (2016). The effect of pro-inflammatory cytokines on immunophenotype, differentiation capacity and immunomodulatory functions of human mesenchymal stem cells. *Cytokine* 85 51–60. 10.1016/j.cyto.2016.06.003 27288632

[B60] Prasanna S. J. Gopalakrishnan D. Shankar S. R. Vasandan A. B. (2010). Pro-inflammatory cytokines, IFNgamma and TNFalpha, influence immune properties of human bone marrow and wharton jelly mesenchymal stem cells differentially. *PLoS One* 5:e9016. 10.1371/journal.pone.0009016 20126406 PMC2814860

[B61] Premer C. Blum A. Bellio M. A. Schulman I. H. Hurwitz B. E. Parker M. (2015). Allogeneic mesenchymal stem cells restore endothelial function in heart failure by stimulating endothelial progenitor cells. *EBioMedicine* 2 467–475. 10.1016/j.ebiom.2015.03.020 26137590 PMC4485912

[B62] Putra A. Ridwan F. B. Putridewi A. I. Kustiyah A. R. Wirastuti K. Sadyah N. A. C. (2018). The role of TNF-α Induced MSCs on suppressive inflammation by increasing TGF-β and IL-10. *Open Access Maced. J. Med. Sci.* 6 1779–1783. 10.3889/oamjms.2018.404 30455748 PMC6236029

[B63] Rahmat Z. Jose S. Ramasamy R. Vidyadaran S. (2013). Reciprocal interactions of mouse bone marrow-derived mesenchymal stem cells and bv2 microglia after lipopolysaccharide stimulation. *Stem Cell Res. Ther.* 4:12. 10.1186/scrt160 23356521 PMC3706938

[B64] Ren G. Zhang L. Zhao X. Xu G. Zhang Y. Roberts A. I. (2008). Mesenchymal stem cell-mediated immunosuppression occurs via concerted action of chemokines and nitric oxide. *Cell Stem Cell* 2 141–150. 10.1016/j.stem.2007.11.014 18371435

[B65] Salomon B. L. Leclerc M. Tosello J. Ronin E. Piaggio E. Cohen J. L. (2018). Tumor necrosis factor α and regulatory T Cells in oncoimmunology. *Front. Immunol.* 9:444. 10.3389/fimmu.2018.00444 29593717 PMC5857565

[B66] Shamdani S. Uzan G. Naserian S. (2020). TNFα-TNFR2 signaling pathway in control of the neural stem/progenitor cell immunosuppressive effect: different experimental approaches to assess this hypothetical mechanism behind their immunological function. *Stem Cell Res. Ther.* 11:307. 10.1186/s13287-020-01816-2 32698887 PMC7374874

[B67] Shen C. Lie P. Miao T. Yu M. Lu Q. Feng T. (2015). Conditioned medium from umbilical cord mesenchymal stem cells induces migration and angiogenesis. *Mol. Med. Rep.* 12 20–30. 10.3892/mmr.2015.3409 25739039 PMC4438972

[B68] Szabó E. Fajka-Boja R. Kriston-Pál É Hornung Á Makra I. Kudlik G. (2015). Licensing by inflammatory cytokines abolishes heterogeneity of immunosuppressive function of mesenchymal stem cell population. *Stem Cells Dev.* 24 2171–2180. 10.1089/scd.2014.0581 26153898

[B69] Tan J. Weil B. R. Abarbanell A. M. Wang Y. Herrmann J. L. Dake M. L. (2010). Ablation of TNF-alpha receptors influences mesenchymal stem cell-mediated cardiac protection against ischemia. *Shock* 34 236–242. 10.1097/SHK.0b013e3181d75ae3 20160664

[B70] Tan K. Zhu H. Zhang J. Ouyang W. Tang J. Zhang Y. (2019). CD73 expression on mesenchymal stem cells dictates the reparative properties via its anti-inflammatory activity. *Stem Cells Int.* 2019:8717694. 10.1155/2019/8717694 31249602 PMC6525959

[B71] Thiagarajan L. Abu-Awwad H. A.-D. M. Dixon J. E. (2017). Osteogenic programming of human mesenchymal stem cells with highly efficient intracellular delivery of RUNX2. *Stem Cells Transl. Med.* 6 2146–2159. 10.1002/sctm.17-0137 29090533 PMC5702512

[B72] Ticha O. Moos L. Wajant H. Bekeredjian-Ding I. (2017). Expression of tumor necrosis factor receptor 2 characterizes TLR9-Driven formation of interleukin-10-producing B Cells. *Front. Immunol.* 8:1951. 10.3389/fimmu.2017.01951 29403470 PMC5780339

[B73] Trivanović D. Krstić J. Djordjević I. O. Mojsilović S. Santibanez J. F. Bugarski D. (2016). The roles of mesenchymal stromal/stem cells in tumor microenvironment associated with inflammation. *Mediators Inflamm.* 2016:7314016. 10.1155/2016/7314016 27630452 PMC5007366

[B74] Vaculik C. Schuster C. Bauer W. Iram N. Pfisterer K. Kramer G. (2012). Human dermis harbors distinct mesenchymal stromal cell subsets. *J. Invest. Dermatol.* 132(3 Pt 1), 563–574. 10.1038/jid.2011.355 22048731 PMC3278768

[B75] Vigo T. Procaccini C. Ferrara G. Baranzini S. Oksenberg J. R. Matarese G. (2017). Uccelli, A. IFN-γ orchestrates mesenchymal stem cell plasticity through the signal transducer and activator of transcription 1 and 3 and mammalian target of rapamycin pathways. *J. Allergy Clin. Immunol.* 139 1667–1676. 10.1016/j.jaci.2016.09.004 27670240

[B76] Wajant H. Siegmund D. (2019). TNFR1 and TNFR2 in the control of the life and death balance of macrophages. *Front. Cell Dev. Biol.* 7:91. 10.3389/fcell.2019.00091 31192209 PMC6548990

[B77] Wang M. Crisostomo P. R. Herring C. Meldrum K. K. Meldrum D. R. (2006). Human progenitor cells from bone marrow or adipose tissue produce VEGF, HGF, and IGF-I in Response to TNF by a P38 MAPK-dependent mechanism. *Am. J. Physiol. Regul. Integr. Comp. Physiol.* 291 R880–R884. 10.1152/ajpregu.00280.2006 16728464

[B78] Watt S. M. Gullo F. van der Garde M. Markeson D. Camicia R. Khoo C. P. (2013). The angiogenic properties of mesenchymal Stem/Stromal cells and their therapeutic potential. *Br. Med. Bull.* 108 25–53. 10.1093/bmb/ldt031 24152971 PMC3842875

[B79] Xu L. Zhou J. Liu J. Liu Y. Wang L. Jiang R. (2017). Different angiogenic potentials of mesenchymal stem cells derived from umbilical artery. Umbilical Vein, and Wharton’s Jelly. *Stem Cells Int.* 2017:3175748. 10.1155/2017/3175748 28874910 PMC5569878

[B80] Yamamoto N. Oyaizu T. Enomoto M. Horie M. Yuasa M. Okawa A. (2020). VEGF and BFGF induction by nitric oxide is associated with hyperbaric oxygen-induced angiogenesis and muscle regeneration. *Sci. Rep.* 10:2744. 10.1038/s41598-020-59615-x 32066777 PMC7026099

[B81] Yan L. Zheng D. Xu R.-H. (2018). Critical role of tumor necrosis factor signaling in mesenchymal stem cell-based therapy for autoimmune and inflammatory diseases. *Front. Immunol.* 9:1658. 10.3389/fimmu.2018.01658 30079066 PMC6062591

[B82] Yang H.-M. Song W.-J. Li Q. Kim S.-Y. Kim H.-J. Ryu M.-O. (2018). Canine mesenchymal stem cells treated with TNF-α and IFN-γ enhance anti-inflammatory effects through the COX-2/PGE2 Pathway. *Res. Vet. Sci.* 119 19–26. 10.1016/j.rvsc.2018.05.011 29783120

[B83] Yang K.-Q. Liu Y. Huang Q.-H. Mo N. Zhang Q.-Y. Meng Q.-G. (2017). Bone marrow-derived mesenchymal stem cells induced by inflammatory cytokines produce angiogenetic factors and promote prostate Cancer Growth. *BMC Cancer* 17:878. 10.1186/s12885-017-3879-z 29268703 PMC5740893

[B84] Yang S. Wang J. Brand D. D. Zheng S. G. (2018). Role of TNF-TNF Receptor 2 Signal in Regulatory T cells and its therapeutic implications. *Front. Immunol.* 9:784. 10.3389/fimmu.2018.00784 29725328 PMC5916970

[B85] Yoshida S. Ono M. Shono T. Izumi H. Ishibashi T. Suzuki H. (1997). Involvement of Interleukin-8, vascular endothelial growth factor, and basic fibroblast growth factor in tumor necrosis factor alpha-dependent angiogenesis. *Mol. Cell. Biol.* 17 4015–4023. 10.1128/mcb.17.7.4015 9199336 PMC232254

[B86] Yu L. Wu Y. Liu J. Li B. Ma B. Li Y. (2018). 3D culture of bone marrow-derived mesenchymal stem cells (BMSCs) could improve bone regeneration in 3D-printed porous Ti6Al4V scaffolds. *Stem Cells Int.* 2018:2074021. 10.1155/2018/2074021 30254680 PMC6145055

[B87] Zhang A. Wang Y. Ye Z. Xie H. Zhou L. Zheng S. (2010). Mechanism of TNF-α-induced migration and hepatocyte growth factor production in human mesenchymal stem cells. *J. Cell. Biochem.* 111 469–475. 10.1002/jcb.22729 20533298

[B88] Zhang C. Lin Y. Liu Q. He J. Xiang P. Wang D. (2020). Growth differentiation factor 11 promotes differentiation of MSCs into endothelial-like cells for angiogenesis. *J. Cell. Mol. Med.* 24 8703–8717. 10.1111/jcmm.15502 32588524 PMC7412688

[B89] Zhang J. Chan Y.-C. Ho J. C.-Y. Siu C.-W. Lian Q. Tse H.-F. (2012). Regulation of cell proliferation of human induced pluripotent stem cell-derived mesenchymal stem cells via Ether-à-Go-Go 1 (HEAG1) potassium channel. *Am. J. Physiol. Cell Physiol.* 303 C115–C125. 10.1152/ajpcell.00326.2011 22357737

[B90] Zhang T. Lee Y. W. Rui Y. F. Cheng T. Y. Jiang X. H. Li G. (2013). Bone marrow-derived mesenchymal stem cells promote growth and angiogenesis of breast and prostate tumors. *Stem Cell Res. Ther.* 4:70. 10.1186/scrt221 23763837 PMC3707041

[B91] Zhang Y. Chiu S. Liang X. Gao F. Zhang Z. Liao S. (2015). Rap1-mediated nuclear factor-KappaB (NF-K B) activity regulates the paracrine capacity of mesenchymal stem cells in heart repair following infarction. *Cell Death Discov.* 1:15007. 10.1038/cddiscovery.2015.7 27551443 PMC4981000

[B92] Zhang Y. Liang X. Lian Q. Tse H.-F. (2013). Perspective and challenges of mesenchymal stem cells for cardiovascular regeneration. *Expert Rev. Cardiovasc. Ther.* 11 505–517. 10.1586/erc.13.5 23570363

[B93] Zhang Y. Yu Z. Jiang D. Liang X. Liao S. Zhang Z. (2016). IPSC-MSCs with High Intrinsic MIRO1 and Sensitivity to TNF-α Yield efficacious mitochondrial transfer to rescue anthracycline-induced cardiomyopathy. *Stem Cell Rep.* 7 749–763. 10.1016/j.stemcr.2016.08.009 27641650 PMC5063626

[B94] Zhou S. Greenberger J. S. Epperly M. W. Goff J. P. Adler C. Leboff M. S. (2008). Age-related intrinsic changes in human bone-marrow-derived mesenchymal stem cells and their differentiation to osteoblasts. *Aging Cell* 7 335–343. 10.1111/j.1474-9726.2008.00377.x 18248663 PMC2398731

[B95] Zhou Y. Tsai T.-L. Li W.-J. (2017). Strategies to retain properties of bone marrow-derived mesenchymal stem cells ex vivo. *Ann. N. Y. Acad. Sci.* 1409 3–17. 10.1111/nyas.13451 28984359 PMC5730506

