# Corrigendum: TNFR2 Is a Crucial Hub Controlling Mesenchymal Stem Cell Biological and Functional Properties

**DOI:** 10.3389/fcell.2021.753407

**Published:** 2021-08-24

**Authors:** Ghada Beldi, Sheyda Bahiraii, Chloé Lezin, Mahsa Nouri Barkestani, Mohamed Essameldin Abdelgawad, Georges Uzan, Sina Naserian

**Affiliations:** ^1^INSERM UMR-S-MD 1197, Hôpital Paul Brousse, Villejuif, France; ^2^Department of Pharmacognosy, University of Vienna, Vienna, Austria; ^3^Paris-Saclay University, Villejuif, France; ^4^Biochemistry Division, Chemistry Department, Faculty of Science, Helwan University, Cairo, Egypt; ^5^CellMedEx, Saint-Maur-des-Fossés, France

**Keywords:** mesenchymal stem cells, immunoregulation, immune-checkpoint, tissue regeneration, angiogenesis, TNFα-TNFR2 signaling pathway

In the original article, there were two mistakes in the legend for **Figure 5** and in Figure 7A.

Regarding the legend for **Figure 5**, we neglected to mention that the graphical images in **Figure 5A** were created with BioRender.com. The correct version appears as below.

**Figure 5**. The TNFR2 Expression by mesenchymal stem cells (MSCs) is associated with the induction of Tregs with more immunosuppressive effect. **(A)** T cells were freshly isolated and depleted from CD25 subpopulation in order to eliminate natural Tregs and highly activated T cells. CD3^+^CD25^−^ Tconvs (orange population) were then added to wild-type (WT) and TNFR2 knockout (KO)-MSCs in a fixed 1/10 MSC/T cell ratio. After 72 h, CD4^+^CD25^+^Foxp3^+^ induced regulatory T cells (iTregs) generated in those co-cultures (green population) were put in an mixed lymphocyte reaction (MLR) test with newly isolated and activated mouse CFSE^+^CD3^+^CD25^−^ Tconvs in a fixed 1/5 iTreg/Tconv ratio. Then, the CD4^+^ and CD8^+^ proliferation capacity was measured by fluorescence-activated cell sorting (FACS). **(B)** Percentage of proliferation of CD4^+^ and CD8^+^ Tconvs in the presence of MSC induced Foxp3^+^ Tregs. Control groups consist of unstimulated T cells alone as depicted by the white columns (*n* = 6), while the stimulated T cells alone are depicted by the black columns (*n* = 6). The blue columns represent the stimulated T cells co-cultured with iTregs derived from WT-MSCs (*n* = 6), and the red columns represent the stimulated T cells co-cultured with iTregs derived from TNFR2 KO-MSCs (*n* = 6). Results are collected from two independent experiments. The graphical images were created with BioRender.com.

Regarding Figure 7A, the representative images used for control groups i.e., MSCs WT and TNFR2 KO in DMEM medium were both from the MSC WT group. New representative images from proper control groups were added in the corrected version of Figure 7. The corrected Figure 7 appears below.

**Figure 7:**
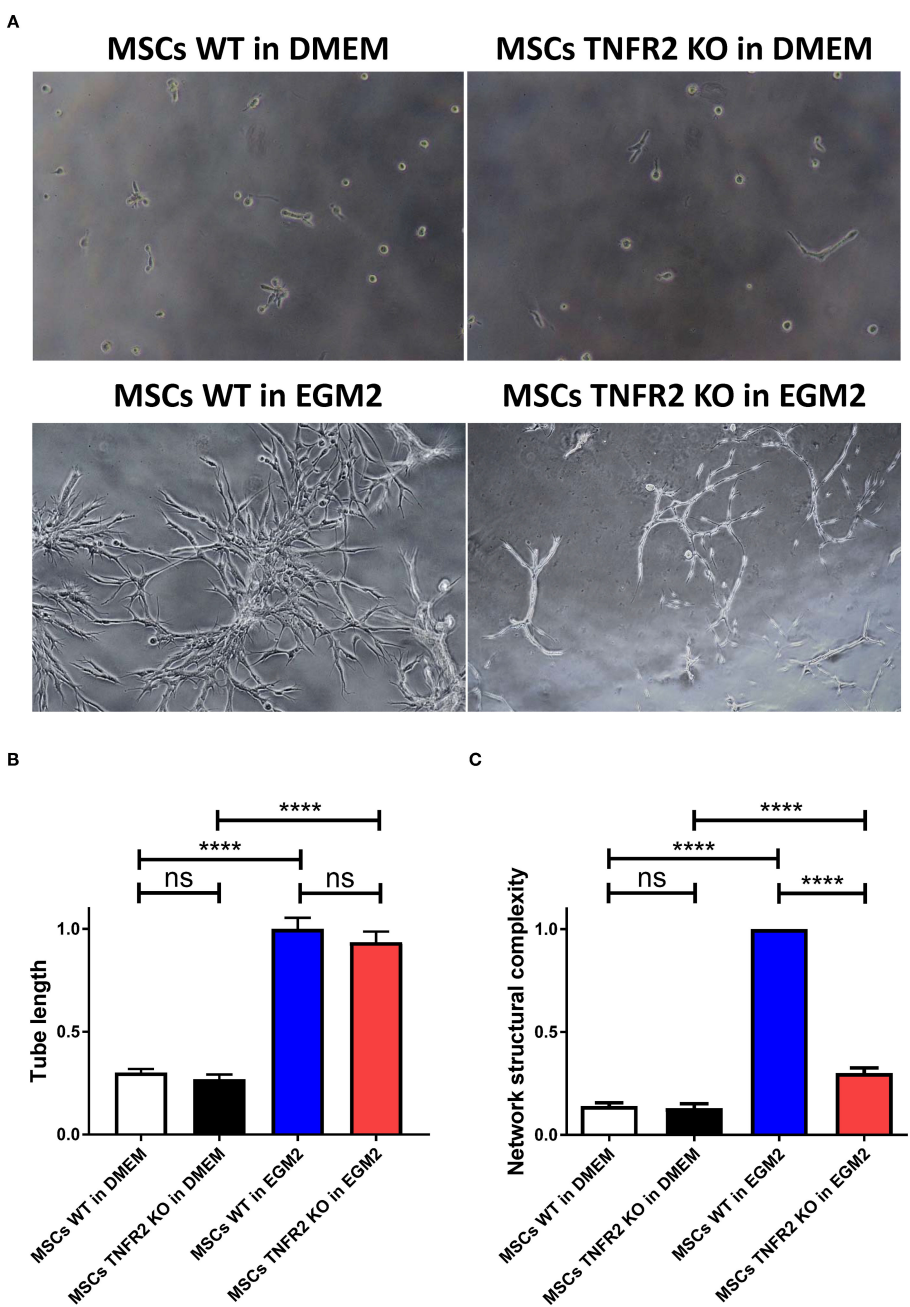
The TNFR2 expression by mesenchymal stem cells (MSCs) is associated with their enhanced tube formation property. To evaluate the involvement of TNFR2 in MSC regenerative feature, wild-type (WT) and TNFR2 knockout (KO)-MSCs (P2 and P3) were cultured on Matrigel using either Dulbecco's modified Eagle's medium (DMEM) standard medium or EGM2 endothelial medium. **(A)** Pictures were taken every 2 h, using objectives 4× and 10× of the inverted microscope in phase-contrast mode. **(B)** The tube length and **(C)** network structural complexity of WT and TNFR2 KO-MSCs were further evaluated. Results are collected from three independent experiments (*n* = 10).

The authors apologize for these errors and state that this does not change the scientific conclusions of the article in any way. The original article has been updated.

## Publisher's Note

All claims expressed in this article are solely those of the authors and do not necessarily represent those of their affiliated organizations, or those of the publisher, the editors and the reviewers. Any product that may be evaluated in this article, or claim that may be made by its manufacturer, is not guaranteed or endorsed by the publisher.

