# Supplementary figures and images for: TNFR2 Is a Crucial Hub Controlling Mesenchymal Stem Cell Biological and Functional Properties
Source: Front Cell Dev Biol. 2020 Dec 4;8:596831. doi: 10.3389/fcell.2020.596831 (PMC7746825; doi:10.3389/fcell.2020.596831)

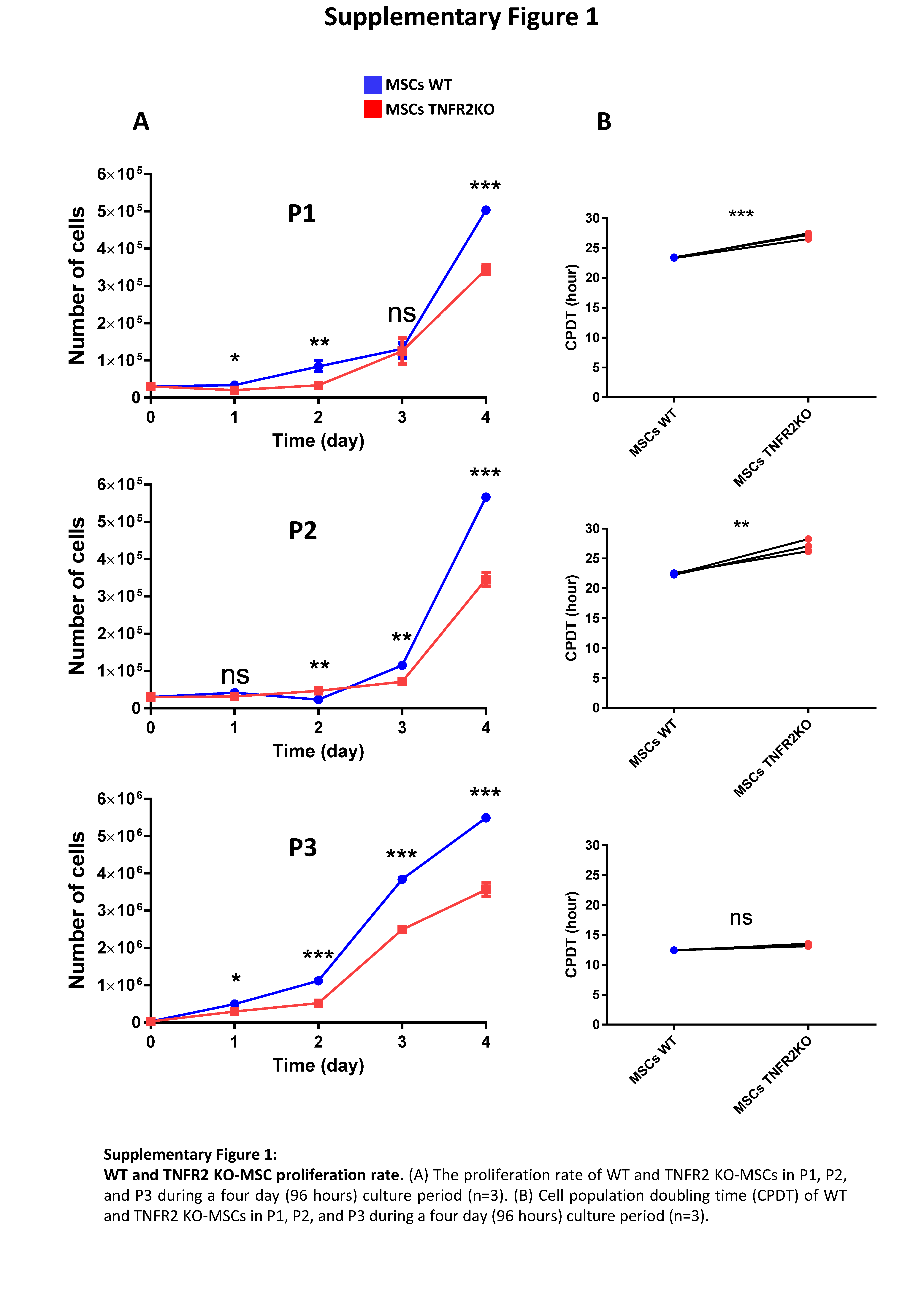

Supplement: Supplementary file 1 [file Image_1.TIFF]

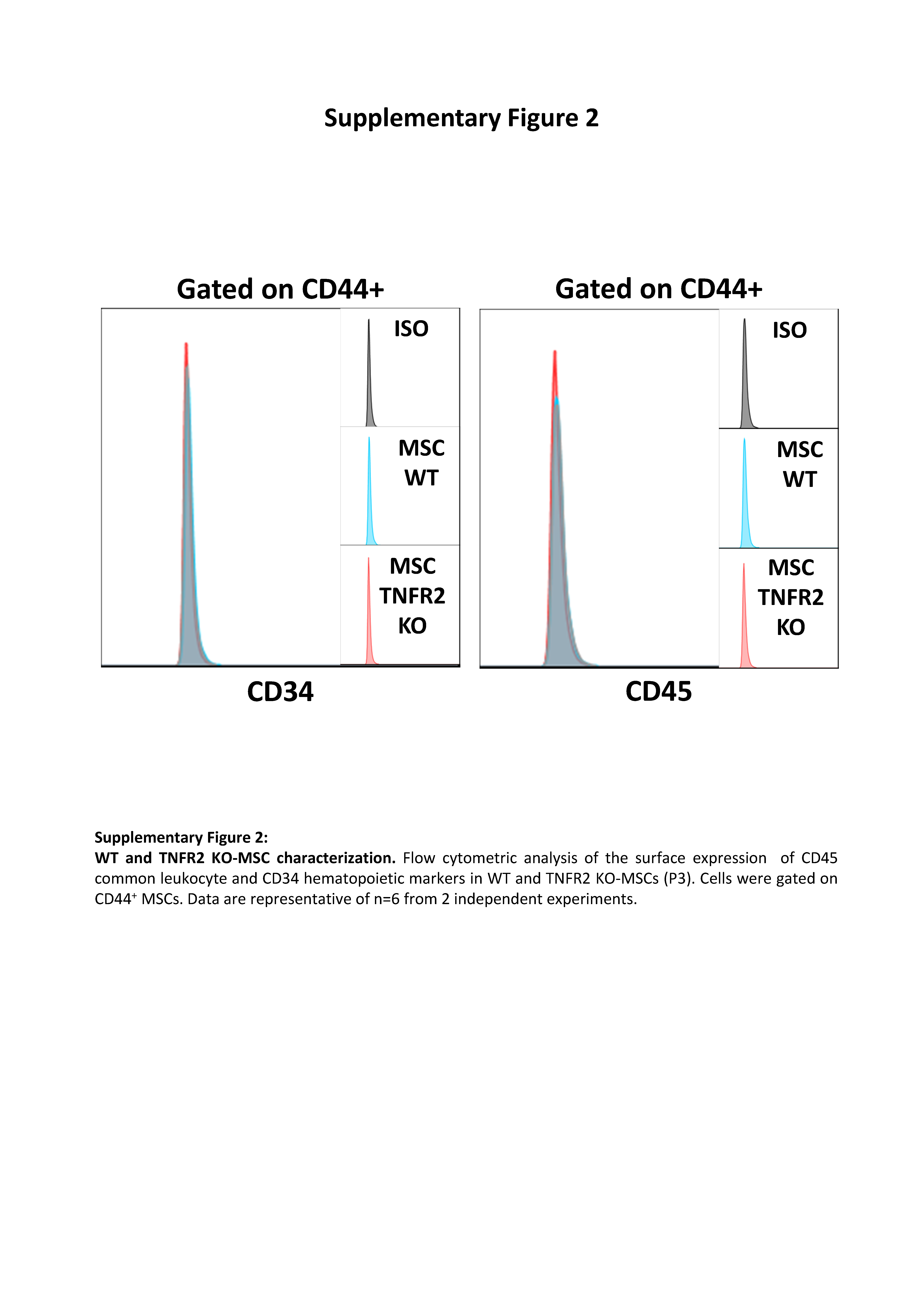

Supplement: Supplementary file 2 [file Image_2.TIFF]
